# Time-resolved multi-omics analysis reveals the role of nutrient stress-induced resource reallocation for TAG accumulation in oleaginous fungus *Mortierella alpina*

**DOI:** 10.1186/s13068-020-01757-1

**Published:** 2020-07-01

**Authors:** Hengqian Lu, Haiqin Chen, Xin Tang, Qin Yang, Hao Zhang, Yong Q. Chen, Wei Chen

**Affiliations:** 1 State Key Laboratory of Food Science and Technology, Jiangnan University, 1800 Lihu Ave, Wuxi, 214122 Jiangsu People’s Republic of China; 2 School of Food Science and Technology, Jiangnan University, Wuxi, 214122 Jiangsu China; 3 National Engineering Research Center for Functional Food, Jiangnan University, Wuxi, 214122 Jiangsu China; 4 (Yangzhou) Institute of Food Biotechnology, Jiangnan University, Yangzhou, 225004 China; 5 Department of Cancer Biology, Wake Forest School of Medicine, Winston-Salem, NC USA; 6 Beijing Innovation Centre of Food Nutrition and Human Health, Beijing Technology and Business University (BTBU), Beijing, 100048 China

**Keywords:** Resource allocation, Lipid biosynthesis, Nutrient stress, Oleaginous microorganism, Metabolic recycling

## Abstract

**Background:**

Global resource reallocation is an established critical strategy through which organisms deal with environmental stress. The regulation of intracellular lipid storage or utilization is one of the most important strategies for maintaining energy homeostasis and optimizing growth. Oleaginous microorganisms respond to nitrogen deprivation by inducing lipid hyper accumulation; however, the associations between resource allocation and lipid accumulation are poorly understood.

**Results:**

Here, the time-resolved metabolomics, lipidomics, and proteomics data were generated in response to nutrient availability to examine how metabolic alternations induced by nitrogen deprivation drive the triacylglycerols (TAG) accumulation in *M. alpina*. The subsequent accumulation of TAG under nitrogen deprivation was a consequence of the reallocation of carbon, nitrogen sources, and lipids, rather than an up-regulation of TAG biosynthesis genes. On one hand, nitrogen deprivation induced the down-regulation of isocitrate dehydrogenase level in TCA cycle and redirected glycolytic flux of carbon from amino acid biosynthesis into fatty acids’ synthesis; on the other hand, nitrogen deprivation induced the up-regulation of cell autophagy and ubiquitin-mediated protein proteolysis which resulted in a recycling of preformed protein nitrogen and carbon. Combining with the up-regulation of glutamate decarboxylase and succinic semialdehyde dehydrogenase in GABA shunt, and the phosphoenolpyruvate carboxykinase in the central hub involving pyruvate/phosphoenolpyruvate/oxaloacetate, the products from nitrogen-containing compounds degradation were recycled to be intermediates of TCA cycle and be shunted toward de novo biosynthesis of fatty acids. We found that nitrogen deprivation increased the protein level of phospholipase C/D that contributes to degradation of phosphatidylcholine and phosphatidylethanolamine, and supplied acyl chains for TAG biosynthesis pathway. In addition, ATP from substrate phosphorylation was presumed to be a critical factor regulation of the global resource allocation and fatty acids’ synthesis rate.

**Conclusions:**

The present findings offer a panoramic view of resource allocation by *M. alpina* in response to nutrient stress and revealed a set of intriguing associations between resource reallocation and TAG accumulation. This system-level insight provides a rich resource with which to explore in-depth functional characterization and gain information about the strategic combination of strain development and process integration to achieve optimal lipid productivity under nutrient stress.

## Background

Organisms ranging from bacteria to mammalian cells use diverse protection mechanisms to adjust metabolism according to environmental stress. These regulatory strategies reflect an inherent trade-off between the benefit and cost of resource investment under stress. Several investigators have recently suggested that cells deal with challenges to growth-limiting perturbations by regulating the allocation of metabolic resources [1–6].

Nutrients serve not only as the resources required by cells to increase mass and generate energy to propel biosynthetic activity, but also as the signals that dictate metabolic, transcriptional, and developmental programs [7, 8]. Cellular responses to nutrient stress have been extensively studied in various model species over many years. One of the most important protection mechanisms is the reallocation of limited carbon, nitrogen, and energy resources to optimize growth and metabolism [7].

Lipids are the storehouses of carbon and energy resources; thus, intracellular lipid storage and utilization are critical for maintaining cellular metabolic homeostasis and survival [9]. Dynamic regulation of intracellular lipid levels and types is an effective strategy to resist nutrient stress [10, 11]. The cellular response to nutrient limitation at the molecular level is the outcome of complex, orchestrated interactions among transcriptional, translational, posttranslational, and metabolic profiles. However, many elementary issues remain unclear; for example, how much cells adjust their composition to cope with the limiting process(es) in response to nutrient limitation and the connection between global resource reallocation and lipid metabolism in response to nutrient stress. Although the effects of nutrient stress on lipid metabolism have been widely studied in various model organisms, consensus has yet to be achieved.

Compelling evidence has suggested that nutrient stress promotes TAG accumulation in various model oleaginous microorganisms [12–21]. Advances in high-throughput measurement technologies have led to progressive investigations into the mechanisms of nutrient stress-induced lipid hyperaccumulation [14, 16, 22, 23]. Recent findings support the notion that oleaginous microorganisms could serve as models to elucidate the resource allocation response to nutrient stress and its association with lipid metabolism. The oleaginous filamentous fungus *M. alpina* is an optimal organism for studying lipid metabolism because not only can it be easily cultured and harvested, but also it can accumulate > 50% of its cell mass as lipids under nitrogen limitation [24, 25]. Our previous genome analysis showed that *M. alpina* harbors the standard components of the desaturation–elongation pathway of long-chain polyunsaturated fatty acids (LC-PUFA) [26]. Furthermore, *M. alpina* is also a promising host for the sustainable generation of high-value LC-PUFA, such as arachidonic (ARA), eicosapentaenoic (EPA), and docosahexaenoic (DHA) acids [27, 28]. Therefore, compared to other oleaginous yeasts or fungi, *M. alpina* as a model species has facilitated the identification of key strategies of resource allocation under nutrient stress and of the critical biosynthetic nodes governing global metabolic flux and lipid biosynthesis. We previously showed that carbon flux, nitrogen metabolism and NADPH generation considerably vary in *M. alpina* under nitrogen limitation [29, 30]. However, understanding the complex interactions involved during resource reallocation at the system level remains limited.

The present bio-physiological study integrated time-resolved phenotypes with multi-omics to quantify global resource allocation by the oleaginous fungus *M. alpina* and determined how *M. alpina* globally allocates resources in response to nutrient stress. The applied strategies can quantitatively and temporally account for all apparent behaviors including changes in the characteristics of bio-physiological parameters and lipid contents throughout fermentation. Our findings will contribute to a deeper understanding of the fundamental mechanisms of cellular responses to nutrient stress, and support the development of new strategies that will achieve more biomass and maximal lipid productivity.

## Results

### Physiological and omics-scale responses of *M. alpina* to nutrient stress

**Fig. 1:**
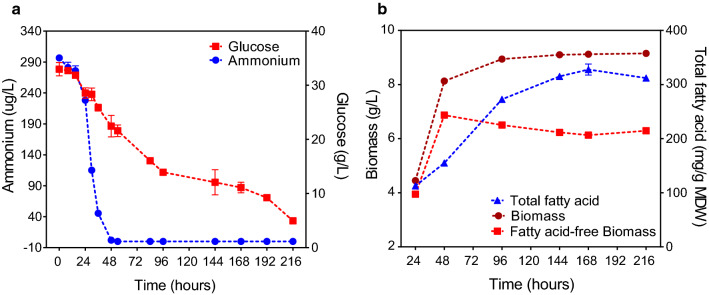
Physiological responses of *M. alpina* to nutrient stress. **a** Changes in carbon and nitrogen concentrations in medium. **b** Change in total fatty acids, biomass (mycelial dry weight), and total fatty acid-free biomass content

Figure 1a shows that the nitrogen content in *M. alpina* cells had decreased within 48 h and was completely exhausted between 36 and 48 h. The extracellular glucose concentration decreased rapidly up to 96 h, and continued to decrease slowly thereafter. This was accompanied by a rapid increase in biomass that peaked with 48 h (Fig. 1b). Intracellular total fatty acids (TFA) accumulated during the early stages of nitrogen deprivation (48–96 h), but then decreased under long-term nitrogen deprivation (96–168 h; Fig. 1b). The amount of fatty acid-free biomass (FFB) was significantly decreased after nitrogen exhaustion and was maintained at the same rate for 168 h (Fig. 1b). There was a close negative correlation between changes in TFA and FFB (*R* = − 0.98) during fermentation under nitrogen deprivation (48–216 h). Taking together, these results indicate that the products of lipid-free substrate degradation induced by nitrogen deprivation play a vital role in fatty acids’ accumulation, especially during the early stages of nitrogen deprivation [31, 32]. The proportion of C20:4 (ARA) decreased after nitrogen deprivation, while that of C18:1 and C18:2 increased significantly (Additional file 1: Table S1).

We applied time-resolved metabolomic and proteomic analyses to *M. alpina* cultured under nutrient stress (Additional file 2). SDS-PAGE results indicate that our prepared protein samples were of adequate quality for subsequent proteomic investigations. Principal component analyses showed that the data points partitioned the samples into the following time-dependent clusters (Additional file 1: Fig. S1): phase 1 (24–48 h), growth, when the biomass rapidly increased; phase 2 (96–168 h), nitrogen deprivation, when the biomass was stable and TFA rapidly accumulated; and phase 3 (216 h), carbon and nitrogen limitation, when TFA decreased and FFB increased.

To determine profiles changes during this time series, we ranked the identified metabolites and proteins using multivariate empirical Bayes analyses (MEBA) (Additional file 3) [33]. The highly ranked metabolites and proteins, in terms of Hotelling T2 statistics, showed that nutrient stress induced a large-scale disturbance in intracellular metabolism (Additional file 1: Fig. S2). Pathway and gene ontology (GO) enrichment analyses using the top 30 Hotelling T2 features indicated that intracellular metabolic pathways and biological processes associated with carbohydrates, amino acids, organic acids, and organic nitrogen were considerably changed during the process of nutrient deprivation-induced lipid accumulation (Additional file 1: Fig. S2e).

**Fig. 2:**
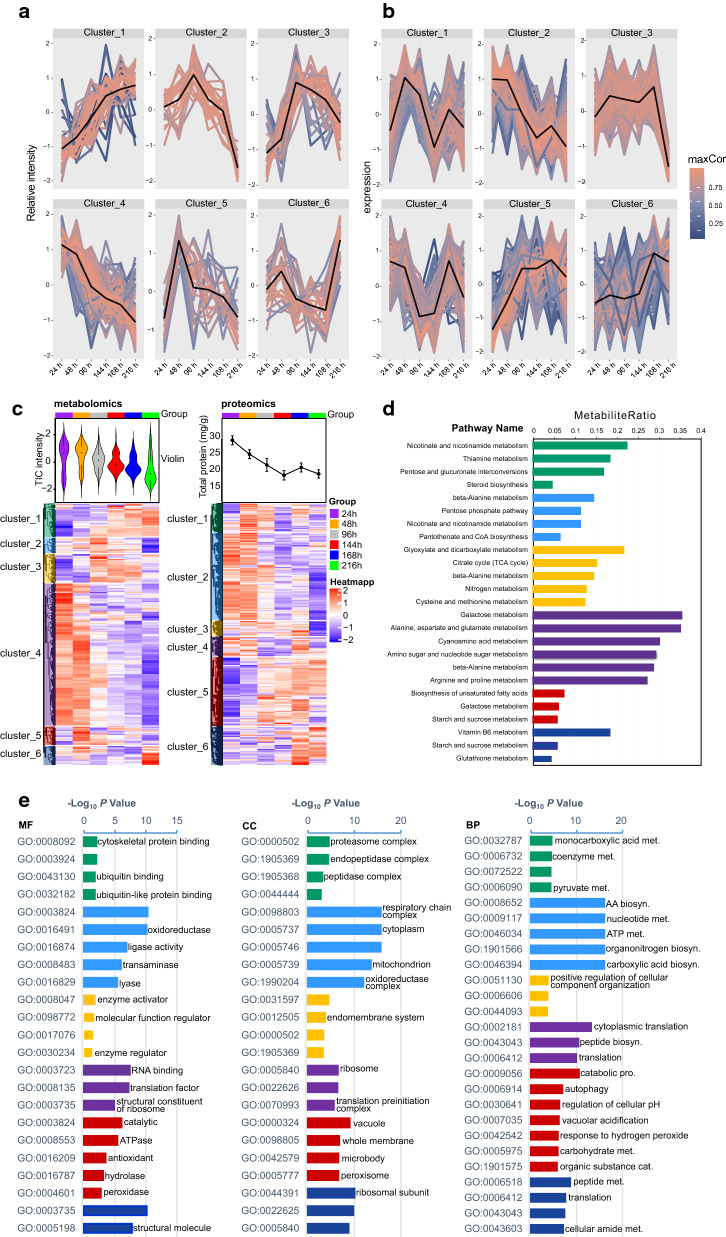
Time-resolved trend clustering analysis. **a**, **b** Cluster of metabolite and protein expression patterns in response to nutrient stress. **c** Six temporal metabolite change/protein expression modules revealed by unsupervised clustering analysis. **d** Top pathways of each cluster from enrichment results. **e** Molecular function (MF), Cellular component (CC) and Biological process (BP) of GO enrichment results of each cluster. Closely correlated metabolites or proteomics (*R*^2^ > 0.75) in each cluster were selected for pathway enrichment or GO enrichment analysis

We used unsupervised time-resolved clustering analyses to quantitatively and temporally elucidate the behavior of differentially altered metabolites and expressed proteins. The results reveal six clusters each of both metabolomics and proteomic datasets (Fig. 2a–c) that were enriched with distinct pathways (Fig. 2d) and GO terms (Fig. 2e). Although the trends in metabolite and protein changes detected herein can be divided into specific modules, the total ion chromatographic (TIC) intensity of polar extracts and total protein content were significantly decreased in *M. alpina* cultured under nutrient stress (Fig. 2c). These results indicate that metabolite and protein profiles are sensitive to the nitrogen and carbon levels in the culture medium, and that their responses are consistent with the timing of the turning point of glucose consumption and TFA accumulation (Figs. 1 and 2a, b).

### Nitrogen deprivation induced carbon metabolism reprogramming

**Fig. 3:**
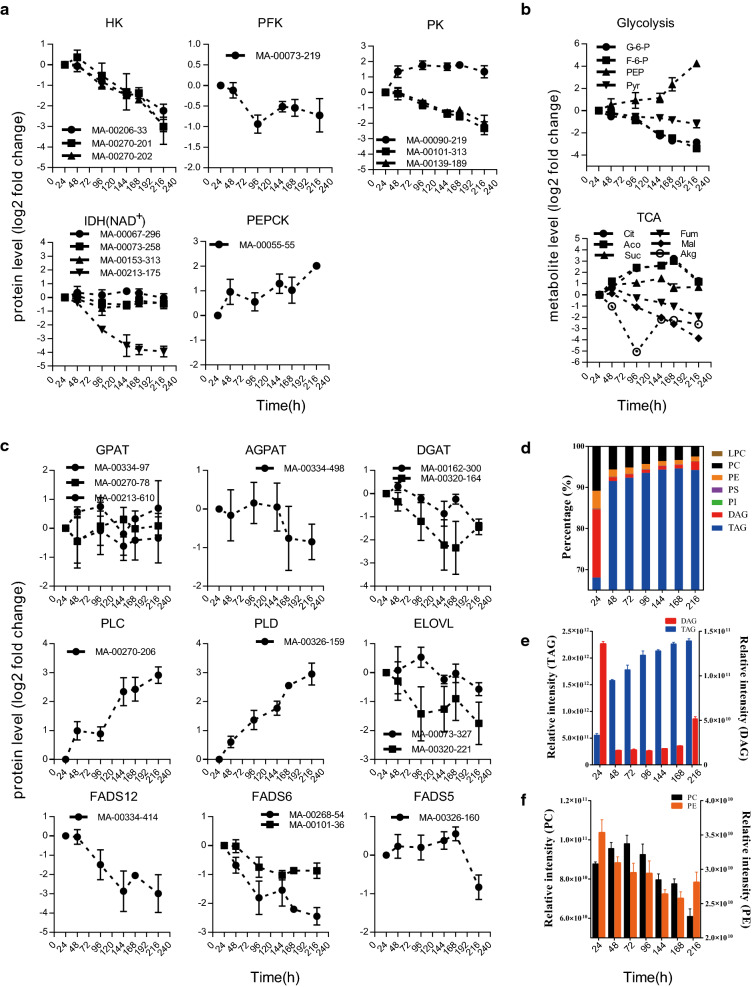
Changes in proteins and metabolites involved in carbon metabolism and lipid metabolism during nitrogen deprivation-induced TAG accumulation. **a** Relative expression of proteins involved in carbon metabolism. **b** Changes in intermediates involved in central carbon metabolism. **c** Relative expression levels of proteins involved in TAG and fatty acid biosynthetic pathways. **d** Ratios of lipid classes. **e** Relative abundances of DAG and TAG. **f** Relative abundances of PC and PE. HK, Hexokinase, PFK, phosphofructokinase, PK, Pyruvate kinase, IDH, Isocitrate dehydrogenase, PEPCK, Phosphoenolpyruvate carboxykinase, GPAT, Dlycero-3-phosphate acyltransferase, AGPAT, 1-acylglycerol-3-phosphate acyltransferase, DGAT, Diacylglycerol acyltransferase, PLC, Phospholipase C, PLD, Phospholipase D, ELOVL, Fatty acid elongase, FADS12, Fatty acid delta 12 desaturase, FADS6, Fatty acid delta 6 desaturase, FADS5, Fatty acid delta 5 desaturase; G-6-P, d-Glucose-6-Phosphate, F-6-P, d-Fructose-6-Phosphate, PEP, Phosphoenolpyruvic acid, Pyr, Pyruvic acid, E-4-P, Erythrose-4-Phosphate, Cit, Citric acid, Aco, cis-aconitic acid, Suc, succinic acid, Fum, fumaric acid, Mal, malic acid, Akg, Alpha-ketoglutaric acid, TAG, triglyceride, DAG, diglyceride, PC, phosphatidylcholine, PE, phosphatidylethanolamine, PI, phosphatidylinositol, PS, Phosphatidylserine, LPC, lysophosphatidylcholine

Integrated metabolomic-proteomic analyses revealed widespread and dynamic remodeling of metabolism that was accompanied by a global metabolic reallocation of resources during TAG accumulation induced by nitrogen deprivation. We found that the rate-limiting enzymes in glycolysis, namely hexokinase (HK), phosphofructokinase (PFK), and pyruvate kinase (PK), were decreased under nitrogen deprivation (Fig. 3a), together with changing trends in the intermediates, glucose-6-phosphate (G-6-P) and fructose-6-phosphate (F-6-P) (Fig. 3b). These findings indicated that nitrogen deprivation induces the downregulation of glycolysis (Additional file 1: Fig. S3).

Most of the enzymes involved in the TCA cycle, especially isocitrate dehydrogenase (IDH), were rapidly decreased in response to nitrogen deprivation (Fig. 3a). However, changes in protein levels did not correlate with changes in the abundance of TCA cycle intermediates (Fig. 3a, b, Additional file 1: Fig. S4). Levels of citric and aconitic acids continuously increased as IDH and aconitase (ACO) decreased in response to nitrogen deprivation, which resulted in a further decrease in intermediates downstream of citric acid (Fig. 3, Additional file 1: Fig. S4). However, succinic acid levels rapidly increased before 144 h (Fig. 3b), during which time other pathways likely supply the carbon skeleton for the succinic acid synthesis under nitrogen deprivation.

Phosphoenolpyruvate (PEP) level was significantly increased during fermentation (Fig. 3b), even though glycolysis was downregulated. These results confirm that pathways other than glycolysis supply the carbon skeleton for the central hub, and involve pyruvate, phosphoenolpyruvate, and oxaloacetate (OAA) under nitrogen deprivation. Phosphoenolpyruvate carboxykinase (PEPCK) level, which plays a key role in gluconeogenesis (a pathway connects TCA cycle and glycolysis), was increased throughout the fermentation process, whereas malate dehydrogenase (MDH) and malic enzyme (ME), which are involved in the pyruvic acid–citric acid cycle, were downregulated (Additional file 1: Figs. S3 and S4). These results indicate that PEPCK rather than MDH/ME plays a vital role in intracellular carbon skeleton recycling, which causes an increase in PEP and stabilizes pyruvic acid levels after downregulation of glycolysis. In *M. alpina*, nitrogen deprivation induces metabolic reprogramming of central carbon metabolism to utilize carbon skeletons that enter the TCA cycle, leading to an accumulation of citric acid for fatty acids’ synthesis.

### Recycling membrane lipids into TAG was active under nitrogen deprivation

Proteomic findings show that enzymes involved in TAG and fatty acid biosynthesis pathways were downregulated or slightly altered under nitrogen deprivation, while phospholipases C (PLC) and D (PLD) which catalyze phosphatidylcholine (PC) and phosphatidylethanolamine (PE) into TAG in the biosynthesis pathway were significantly increased throughout the fermentation process (Fig. 3c). Lipidomic results show that TAG levels continued to increase after 48 h independently of cell growth (Fig. 3d, e), suggesting that nitrogen deprivation induces TAG biosynthesis. Notably, the ratios (%) and contents of membrane lipids (PC and PE) decreased with increasing TAG within 168 h (Fig. 3d, e, f), which is consistent with changes in PLC and PLD protein level. We ranked the detected TAG and found that the length and number of unsaturated bonds in TAG changed from low to high during fermentation (Additional file 1: Fig. S5).

### Ubiquitin-mediated proteolysis and cell autophagy were upregulated during nitrogen deprivation

**Fig. 4:**
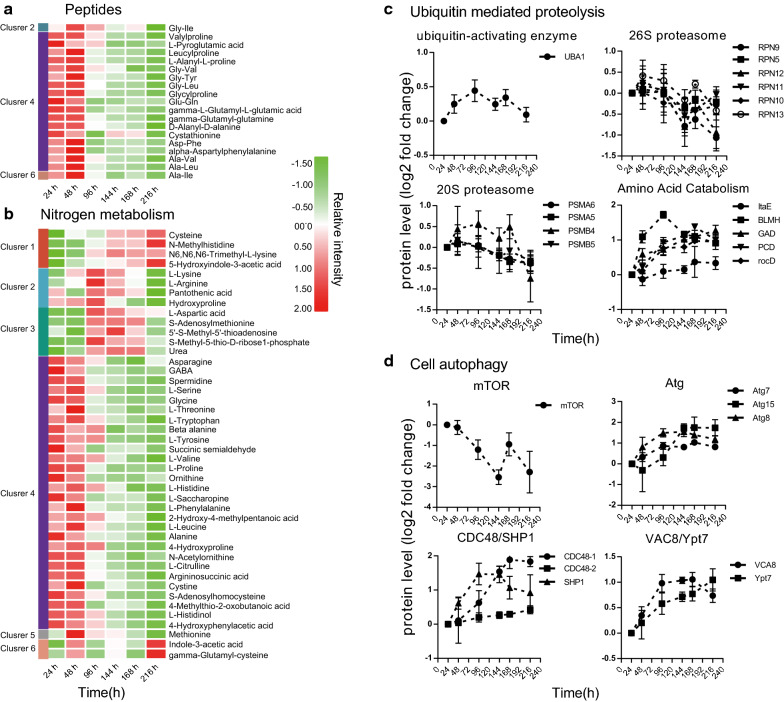
Responses of metabolic recycling systems to nitrogen stress in *M. alpina*. Heatmap shows changes in peptides (**a**) and metabolites (**b**) involved in nitrogen metabolism (especially amino acid metabolism) during fermentation. **c** Changes in enzymes involved in ubiquitin mediated proteolysis pathway. **d** Changes in key enzymes involved in cell autophagy. UBA1, ubiquitin-activating enzyme E1, UBLE1B, ubiquitin-like 1-activating enzyme E1 B, RPN, 26S proteasome regulatory subunit, PSMA, 20S proteasome subunit alpha, PSMB, 20S proteasome subunit beta, ltaE, threonine aldolase, BLMH, bleomycin hydrolase, GAD, glutamate decarboxylase, PCD, 1-pyrroline-5-carboxylate dehydrogenase, rocD, ornithine-oxo-acid transaminase, mTOR, serine/threonine-protein kinase mTOR, Atg, autophagy-relate gene, CDC48, transitional endoplasmic reticulum ATPase, SPH1, UBX domain-containing protein 1, VAC8, vacuolar protein 8, Ypt7, GTP-binding protein

Total proteins, peptides, and amino acids decreased rapidly in response to nitrogen deprivation (Fig. 2c; Fig. 4a, b). Furthermore, levels of nitrogen-containing compounds, namely, pyridoxine, pyridoxamine, pyridoxal, adenosine, xanthine, uracil, cytidine, and uridine, which are involved in cofactor and nucleotide metabolism, were also significantly decreased (Additional file 1: Fig. S6). However, levels of l-arginine and urea, two critical intermediates involved in the urea cycle, were increased after nitrogen exhaustion (Fig. 4b).

Our time-resolved trend analysis identified enriched ubiquitin-like protein binding in cluster 1 (Fig. 2e). The 26S proteasome regulatory subunits (RPN; RPN5, RPN7, RPN9, RPN10, RPN11, RPN12, RPN13, PSMA5, PSMB5, and PSMA6), which are the enzymes involved in the ubiquitin–proteasome protein degradation system (UPS), were increased or stable during the early stages of nitrogen deprivation and began to decrease after 96 h (Fig. 4c). Ubiquitin-activating enzyme E1 (UBA1), which is involved in the UPS, was upregulated when nitrogen was exhausted (Fig. 4c). We further analyzed enzymes involved in cellular amino acid catabolism from cluster 5 and found that bleomycin hydrolase (BLMH), threonine aldolase (ltaE), 1-pyrroline-5-carboxylate dehydrogenase (PCD), glutamate decarboxylase (GAD), and ornithine-oxo-acid transaminase (rocD) were upregulated (Fig. 4c). These proteins are mainly involved in glycine, serine and threonine, alanine, aspartate and glutamate, arginine and proline, and beta-alanine metabolism.

Results from the present study found that nitrogen deprivation induced a significant downregulation of mammalian target of rapamycin (mTOR) protein (Fig. 4d), which is an important negative regulator of cellular autophagy. In addition, GO enrichment analysis identified autophagy as one of the top GO items in cluster 5 (Fig. 2e), in which protein abundance increased continuously and showed a positive correlation with fatty acid synthesis during fermentation. Taking together, all these findings indicate that autophagy is enhanced under nitrogen deprivation and is likely to be an important biological process for efficient TAG accumulation. We surveyed the biological process items of cluster 5 and found autophagy, lysosomal microautophagy, macroautophagy, and autophagy of the nucleus (Additional file 4: cluster 5). In addition, aging has been reported to be important for fatty acid synthesis, and always occurs together with autophagy [34]. We selected proteins in cluster 5 related to each autophagy-related biological process, including aging, and represent their overlap in a Venn diagram (Additional file 1: Fig. S7). Eight proteins were shared among the four autophagy-related biological processes, and nine proteins were uniquely associated with autophagy and aging. Only one protein was shared between aging and other autophagy-related biological processes, suggesting that lipid accumulation affected by aging may proceed via autophagy-independent machinery. The eight shared proteins are involved in protein processing in the endoplasmic reticulum (CDC48/SHP1), the cytoplasm to vacuolar targeting pathway (VAC8), autophagosome biogenesis and elongation (CDC48-SHP1/Atg8), and vacuole fusion (Ypt7/Atg15)(Fig. 4d). Autophagy-related proteins, including Atg7, Atg8, and Atg15, which are important for autophagosome biogenesis, were increased in response to nitrogen deprivation (Fig. 4d).

### Survey of protein expression at the subcellular level

Gene ontology enrichment of the highly correlated proteins shown in Fig. 2e revealed a significant over-representation of numerous “cellular component (CC)” terms. These proteins are associated with mitochondrial, peroxisomal, vacuolar, and whole membranes (Additional file 5) and are involved in carbon metabolism, energy metabolism, protein processing, fatty acid oxidation, antioxidation, and cell autophagy.

**Fig. 5:**
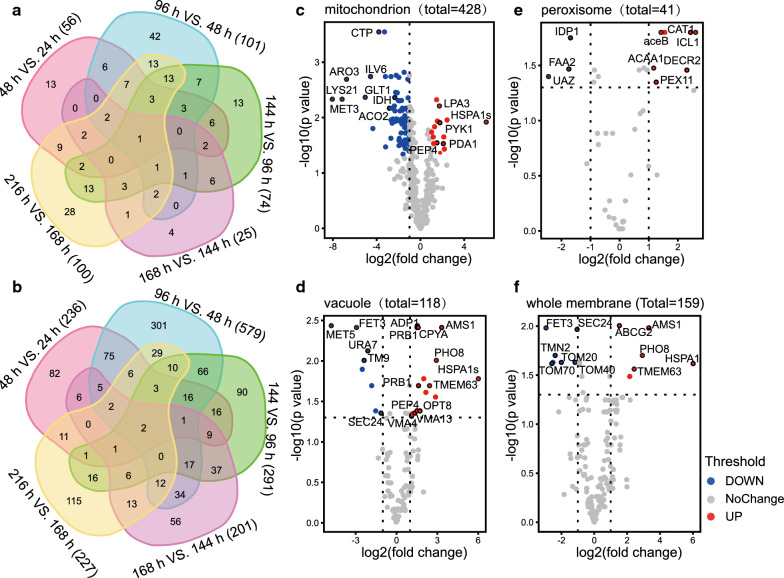
Impact of nitrogen deprivation on cellular compartment abundance in *M. alpina*. Venn diagram shows overlap in numbers of metabolites (**a**) and proteins (**b**) that were significantly upregulated or downregulated. **c**–**f** Volcano plots show changes in proteins assigned by gene ontology to specific compartments at 96 h vs. 24 h (Log 2 FC > 2, *p* < 0.05). Protein abundance was determined using tandem mass spectrometry and quantified by MS1 precursor ion intensity. Protein annotations are described in detail in Additional file 5

The Venn diagram shows that the number of significantly changed metabolites and proteins peaked at 48–96 h, and the most significantly changed metabolites/proteins at 96 h vs. 48 h did not undergo further significant changes during the remainder of the fermentation process (Fig. 5a, b). To further parse the effects of nitrogen deprivation on specific compartments within the *M. alpina* proteomics, we individually analyzed specific GO subcategories using volcano plots and compared the protein profiles from 24 to 96 h (Fig. 5c–f). Our results indicate that most of the mitochondria-associated proteins were downregulated whereas those associated with vacuoles were upregulated, in response to nitrogen deprivation (Fig. 5c, d). The significantly decreased proteins in mitochondria, ARO3, LYS21, ILV6, and GLT1, are involved in amino acid biosynthesis (Fig. 5c). The significant downregulation enzymes involved in TCA, IDH, and ACO2 could explain the significant accumulation of citric acid under nitrogen deprivation (Figs. 3b and 5c). Pyruvate kinase 1 (PYK1) and the pyruvate dehydrogenase complex (PDA1), which catalyze the conversion of phosphoenolpyruvate/pyruvate to acetyl-CoA, were upregulated under nitrogen deprivation (Fig. 5c). This contributes to recycling and fluxing of the intracellular carbon skeleton into the TCA cycle for fatty acid synthesis. The upregulated mitochondria proteins (LPA3, HSPA1s and PEP4) are involved in protein degradation and cell autophagy (Fig. 5c). Peroxisomal biogenesis factor 11 (PEX11) is an important enzyme that controls peroxisomal division and is upregulated under nitrogen deprivation (Fig. 5e). We found that peroxisomal 2,4-dienoyl-CoA reductase (DECR2) and acetyl-CoA acyltransferase 1 (ACAA1), which play an important role in very long-chain fatty acid oxidation, and catalase (CAT1) was increased under nitrogen deprivation (Fig. 5e) [35]. In addition, malate synthase (aceB) and isocitrate lyase (ICL1), which are involved in the glyoxylate cycle in peroxisomes, were also significantly increased in *M. alpina* under nitrogen deprivation (Fig. 5e). The upregulated proteins HSPAs1, AMS1, OPT8, CPYA, PEP4, VMA13/4, and PRB1 in vacuoles are involved in cell autophagy, including transportation and degradation of proteins/peptides and endocytosis (Fig. 5d). The transport protein, sec24, which belongs to the transmembrane 9 (TM9) superfamily, the cytidine triphosphate (CTP) synthase URA7, the iron transport multicopper oxidase FET3, and the pyruvate flavodoxin/ferredoxin oxidoreductase MET5 were all significantly decreased, indicating that protein biosynthesis and sorting are downregulated under nitrogen deprivation (Fig. 5d). With the exception of the ABC transporter ABCG2, proteins that were significantly increased in vacuoles were also found in whole membranes (Fig. 5d and f), which reveals an important role for transmembrane transport centered on vacuoles. The mitochondrial import receptor subunit (TOM20/40/70) membrane proteins, which are important for mitochondrial biogenesis, were significantly decreased (Fig. 5f). This indicates that mitochondrial activity is downregulated under nitrogen deprivation.

## Discussion

The present study investigated metabolomic and proteomic changes in pathways and biological processes during different fermentation phases in the oleaginous fungus *M. alpina* from the perspective of TAG accumulation. A multidimensional, high-resolution atlas allowed a detailed analysis of changes in metabolites and proteins induced by nutrient stress throughout the fermentation process. This significantly increased the reliability of our interpretation of the relationships between intracellular metabolites and TAG synthesis.

### Carbon and nitrogen reallocation in response to nitrogen deprivation plays key roles in providing precursors for TAG synthesis

**Fig. 6:**
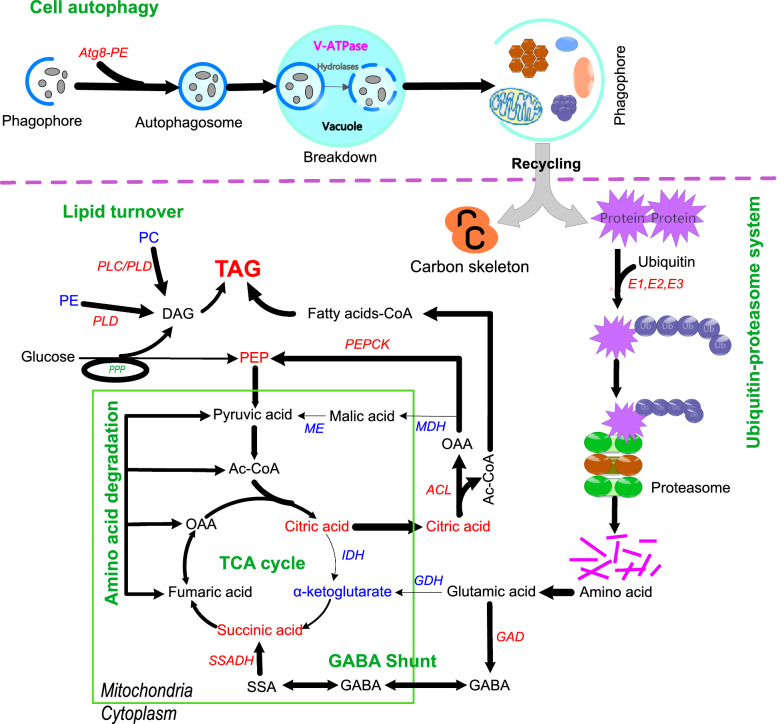
Schematic diagram showing the key connections between resource reallocation and TAG accumulation in *M. alpina* under nitrogen deprivation. Upregulated (red) and downregulated (blue) metabolites and enzymes. MDH, malate dehydrogenase, GDH, glutamate dehydrogenase, SSADH, succinate-semialdehyde dehydrogenase, PLC, Phospholipase C, PLD Phospholipase D, IDH, Isocitrate dehydrogenase, ME, Malic enzyme, ACL, ATP-citrate lyase, PEPCK, Phosphoenolpyruvate carboxykinase; OAA, Oxaloacetic acid, PEP, Phosphoenolpyruvic acid, TAG, triglyceride, DAG, diglyceride, PC, phosphatidylcholine, PE, phosphatidylethanolamine

Compelling evidence has shown that, under nitrogen deprivation, oleaginous microorganisms can utilize excessive extracellular carbon sources for lipid biosynthesis via glycolysis and lipid biosynthesis pathways [19, 25]. Here, we propose that, with the exception of glycolytic flux of carbon sources, the products of intracellular substrate degradation likely serve as an important source of acetyl-CoA for fatty acid synthesis under nitrogen stress [32, 36–39]. In response to nitrogen deprivation, *M. alpina* downregulates biosynthetic metabolism and shunts internal pools of nitrogen toward nitrogen assimilation machinery. Proteins and nucleotides comprise the major sink of nitrogen storage under optimal conditions, whereas nitrogen is scavenged from organic compounds via several catabolic pathways under nitrogen deprivation [40, 41]. The net effect of this intracellular resource reallocation is that cells can quickly respond to changes in external nitrogen availability, thereby maintaining their survival. Nitrogen and carbon metabolisms are closely connected. Our previous genome-scale metabolic model analysis revealed that amino acid degradation is a source of acetyl-CoA for fatty acid synthesis [42]. Thus, the high-intensity metabolism of the proteolysis and oxidation pathways of the amino acid carbon skeleton that occurs in *M.alpina* under nitrogen deprivation could supply two-carbon donor molecules for fatty acid synthesis [37, 43–45]. This is consistent with the findings of Levitan et al., showing that about half of all the intracellular proteins in *Phaeodactylum tricornutum* are degraded under nitrogen deprivation, and then the catabolic products of amino acids flow into the TCA cycle where they can participate in fatty acid biosynthesis [46]. Generally, the intermediate metabolism of carbon and nitrogen in eukaryotic microbes is closely coupled with a hub centered around glutamate and α-ketoglutarate [47]. Glutamate supplementation increases ARA production in *M. alpina* [48, 49]. Thus, glutamate and α-ketoglutarate have always been thought as a hub for recycling carbon skeletons into the TCA cycle. However, results from the current study show that the levels of glutamate dehydrogenase (GDH) and α-ketoglutarate are significantly decreased in response to nitrogen deprivation (Fig. 6). In contrast, the rate-limiting enzymes involved in the γ-amino butyric acid (GABA) shunt, such as glutamate decarboxylase (GAD) and succinic semialdehyde dehydrogenase (SSADH), were significantly increased after the nitrogen source was exhausted (Additional file 1: Fig. S8). Succinic acid, as the final product of the GABA shunt, was increased under nitrogen deprivation, even when the TCA cycle was downregulated. Therefore, rather than the glutamate and α-ketoglutarate pathway, the GABA shunt played a more important role in recycling the carbon skeleton from amino acids into the TCA cycle in *M.alpina*, (Fig. 6). Glutamate supplementation and enhancing the GABA shunt via metabolic engineering may be an efficient strategy for the development of cell factories for hyper lipid production. In addition, the current study revealed that the PEP levels are increased in this study, even though glycolysis is downregulated. We also found that the enzymes involved in the pyruvate-mediated acetyl-CoA pathway are upregulated during fermentation. Therefore, the central hub involving pyruvate, phosphoenolpyruvate, and OAA is upregulated in nitrogen-stressed cells, and this intermediate metabolic pathway is likely to be a significant source of carbon for fatty acid biosynthesis (Fig. 6). Previous work indicated that recycling of OAA into glycolysis is mediated by malic acid, and that the NADPH yield is the major source of NADPH required for fatty acid synthesis [50]. In *M.alpina*, however, PEPCK, rather than MDH/ME, plays a vital role in intracellular carbon skeleton recycling, which causes an increase in PEP and stabilizes pyruvic acid levels (Fig. 6). Therefore, regulation of the carbon flow between PEPCK and the MDH/ME pathway via metabolic engineering could provide a more rational supply of both the carbon skeleton and NADPH, which could be an efficient strategy for hyper fatty acid production (Fig. 6). Branched-chain amino acid degradation is thought to direct carbon and energy toward TAG accumulation under nitrogen deprivation [37, 43, 51]. The present study found that supplementing nitrogen-limited culture medium with l-valine significantly increased the content of odd-chain fatty acids in *M. alpina* (Additional file 1: Fig. S8d), indicating the ability of this organism to convert l-valine to odd-chain fatty acids under nitrogen stress [52, 53].

Perhaps the most noteworthy change in the lipid profiles of nitrogen-stressed *M. alpina* was the increase in TAG accumulation and diversity, which was paralleled by a decrease in membrane lipids (PC and PE). The role of membrane lipid turnover in TAG synthesis has been widely investigated [12, 39]. In the current study, PLC and PLD, which catalyze the conversion of PC and PE into the TAG biosynthesis pathway, were found to be significantly increased in response to nitrogen deprivation, and were identified as the key enzymes in membrane lipid turnover. Thus, consistent with previous reports, in *M.alpina*, TAG accumulation in response to nitrogen deprivation is also a consequence of recycling and reallocating acyl chains in different lipid classes [54], rather than the upregulation of lipid biosynthesis genes. Beta-oxidation of LC-PUFA in peroxisomes was also found to be upregulated under nitrogen stress. Fatty acid profiles revealed that the proportion of ARA is significantly decreased during fermentation, while the proportion of C16–C18 fatty acids is increased (Additional file 1: Table S1). Therefore, rather than inhibiting LC-PUFAs synthesis, nitrogen deprivation induces LC-PUFA degradation and provides a carbon skeleton for the synthesis of C16–C18 series fatty acids. Generally, under nitrogen deprivation, up-regulation of the enzymes in fatty acid synthesis and down-regulation of the enzymes involved in fatty acid degradation were found as a common feature in oleaginous microorganisms, while this seems not the case in the current study, that maybe due to the differences of the length of the fatty acid synthesis pathway and the different substrate specificity of enzymes involved in fatty acid synthesis pathway between *M.alpina* and other species [26, 55].

### Importance of ubiquitin-mediated proteolysis and cell autophagy to cellular carbon and nitrogen reallocation

The most notable change in the protein profiles of nitrogen-stressed *M. alpina* is the upregulation of proteolysis and amino acid degradation. UPS is an important protein degradation system in organisms in which proteins are degraded in highly specific ways. UPS and ERAD are coupled to the targeted degradation of damaged or defective proteins [56]. Regardless of whether UPS or ERAD are involved, ubiquitination is the first step in protein degradation, comprises a series of reactions involving ATP-dependent ubiquitin-activating enzyme 1, ubiquitin-conjugating enzyme and ubiquitin protein ligases (Fig. 6) [57–59]. The findings of our trend analyses show that protein ubiquitination is highly active during the early phase of nitrogen deprivation and is inhibited at the later stage (Fig. 2e, cluster 1), which is consistent with changes in the TFA accumulation rate. This reveals a potential correlation between protein ubiquitination and TAG accumulation, and suggests that UBA1 is a key gene modification target used in the regulation of protein ubiquitination levels for efficient lipid accumulation.

Compared with UPS and ERAD, cell autophagy in *M. alpina* is continuously upregulated from nitrogen-replete to nitrogen-deprived (Fig. 2c, cluster 5). Autophagy is also an important bioprocess for delivering cytoplasmic constituents into vacuoles for degradation. This catabolic process enables not only the removal of obsolete or damaged macromolecules, defective organelles, and invasive microorganisms, but also promotes the simultaneous recycling of cellular components into valuable nutrients [59, 60]. Thus it is essential for homeostasis, development, and survival. Compelling evidence supports the notion that cell autophagy is upregulated under nutrient deprivation [61]. Protein and rRNA turnover require core autophagy genes [62], and the findings from the current study are consistent with this. Several assays have been developed to determine the role of autophagy in lipid metabolism [63]. Couse et al. [64] found that autophagy is required for TAG synthesis and the formation of lipid bodies in nitrogen-starved cells, as well as for the degradation of ribosomal proteins in *Chlamydomonas*. Fan et al. [65] showed that basal autophagy contributes to TAG synthesis in *Arabidopsis*, and that disrupted basal autophagy impedes organelle membrane lipid turnover, and hence preventing fatty acid mobilization from membrane lipids to TAG. The lipid profiles of nitrogen-stressed *M. alpina* described in the current study revealed an increased in TAG accumulation, which is paralleled by a decrease in PE and PC. Autophagy also promotes the degradation and disruption of lipid droplets in various species under long-term nutrient deprivation [63, 66, 67]. Transmission electron microscope images of mycelia at the end of the fermentation process revealed that a large proportion of cell bodies are occupied by completely spherical lipid droplets (Additional file 1: Fig. S9), indicating that nitrogen deprivation-induced cell autophagy does not promote lipid droplet degradation in *M. alpina*.

CDC48 and SHP1 are important enzymes for in ER-associated degradation (ERAD). Roswitha Krick et al. found that CDC48 and its substrate-recruiting cofactor SHP1 regulate autophagosome biogenesis in concert with ubiquitin-like Atg8 [68]. Furthermore, nitrogen deprivation induces the continuous up-regulation of CDC48 and SHP1 (Fig. 4d), indicating that ERAD and autophagy play an important role in the reallocation of nitrogen and carbon skeletons for cell survival and fatty acid synthesis during fermentation [67].

### Potential mechanisms affecting TAG synthesis rates

In the current study, TFA content was found to be continuously increasing during the fermentation, but the rate of TFA accumulation decreases after 96 h (Fig. 1). Under nitrogen deprivation, TCA cycle blocking not only shunts carbon skeletons toward fatty acid biosynthesis, but also prevents flavin adenine dinucleotide (FADH2) synthesis. Together with the downregulation of glycolysis, the amounts of FADH2 and nicotinamide adenine dinucleotide (NADH), which are important for ATP synthesis in the electron transfer chain, are decreased. In addition, enzymes involved in oxidative phosphorylation are significantly decreased during fermentation (Additional file 1: Fig. S10). Taken together, these findings can fully explain the continuous decline in ATP levels during fermentation. Low ATP improves glycolysis flow, which is consistent with the rapid decrease in glucose concentration and the rapid increase in fatty acid accumulation between 48 and 96 h, as demonstrated in the present study. Chen et al. [69] postulated that additional energy in the form of ATP is required to drive this biosynthetic process, and that enzymes and structural proteins depend on ATP for motility and as well as to fuel biosynthetic reactions. Here, we found that UBA1 is ATP dependent [70]; therefore, when ATP decreased to a threshold level, it inhibits UBA1 activity and prevents UPS and ERAD, finally resulting in a decreased rate of fatty acid synthesis. Proteases involved in the UPS, such as proteasome complexes and endopeptidase complexes, are also ATP-dependent and are downregulated during the later, rather than early stage. In addition, levels of other ATP-dependent enzymes, such as HK, PFK and ATP citrate lyase (ACL) that play important roles in fatty acid synthesis, were also significantly decreased during the later phase of fatty acid accumulation; this may be due to a serious ATP deficiency. The rate of respiratory oxidative phosphorylation decreases during lipid droplet formation induced by nitrogen deprivation. Cyclic electron flow has been shown to produce the ATP needed for nitrogen deprivation-induced TAG synthesis in green alga [69, 71]. However, we did not find cyclic electron flow in *M. alpina*. Instead, substrate level phosphorylation is the most likely source of ATP that drives lipid accumulation under nutrient deprivation in *M. alpina* [72]. Changes in metabolomic and proteomic profiles indicate vigorous substrate oxygenolysis during the early stage of nitrogen deprivation. However, this declines after 96 h, which could be the reason for differences in the rate of fatty acid synthesis and TAG biosynthesis before and after 96 h. Manipulating ATP supply and demand could therefore a powerful strategy by which *M. alpina* synthesizes more fatty acids.

Our findings revealed that a considerable amount of degradation products from UPS/cell autophagy pathways and membrane lipids is critical metabolites for the provision of fatty acids and/or lipid precursors for TAG synthesis under nitrogen deprivation. However, most of the degradation products, namely amino acids, nucleotides, and intermediates involved in PPP, the GABA shunt, the TCA cycle, and DAG, are significantly decreased. This indicates a state of disequilibrium between supply and demand under nitrogen deprivation, especially after 96 h. *M. alpina* has the ability to convert pre-formed carbon sources into fatty acids under nitrogen deprivation, while substrate restriction limits the efficiency of this important conversion process.

All of this can be explained by an imbalance between biosynthesis and catabolism and can be attributed to the continuous shutting down of the TCA cycle. Thus, a dynamic trade-off in IDH expression level in the TCA cycle using inducible promoters or biosensors could be an efficient strategy for regulating cell biomass and lipid synthesis, thereby assuring that lipids can be continuously synthesized at a high rate.

## Conclusions

We used multivariate empirical Bayes analyses (MEBA), trend cluster and subcellular organelle analysis to elucidate dynamic changes in metabolic and protein profiles throughout fermentation from nitrogen-replete to nitrogen-free conditions. We found that nitrogen deprivation induced TAG accumulation as a consequence of nitrogen and carbon reallocation mediated by ubiquitination modification and autophagy. The TCA cycle is a hub of carbohydrate, lipid, and amino acid metabolism. When nitrogen availability decreases, the sink for TCA cycle metabolites can be shunted toward fatty acid biosynthesis through the downregulation of IDH, which blocks the TCA cycle. Therefore, intracellular protein content decreases, whereas storage lipids increase under nitrogen stress. We also uncovered some factors produced under long-term nitrogen deprivation that could limit the efficient biosynthesis of TAG, such as imbalanced energy metabolism, the beta-oxidation of LC-PUFA in peroxisomes. Our results showed that *M. alpina* can remodel its intracellular metabolism temporally to respond to changes in environmental cues during fermentation, and revealed the key hubs, metabolic pathways and biological processes governing this resource allocation, which played an important role in fatty acid accumulation. The present findings revealed a global metabolic landscape of resource allocation by *M. alpina* in response to nutrient stress, and the relationship between such allocation and TAG accumulation. The insights afforded here provide an important reference for the development of more efficient engineering strains and diverse control strategies for smart fermentation processes to produce microbe-based functional oils.

## Methods

### Chemicals, strain, media, and cultivation conditions

All HPLC-grade solvents and standards were purchased from Merck KGaA (Darmstadt, Germany) and Sigma-Aldrich Corp. (St. Louis, MO, USA) unless otherwise noted. The *M. alpina* strain (ATCC322222, American Type Culture Collection, Manassas, VA, USA) was cultured as we described in our previous publications [29] and in Additional file 6. Nitrogen stress studies proceeded in 7.5 L-fermentation tanks containing 4.0 L Kendrick broth with ammonia as the nitrogen source. Fresh mycelia and fermentation broth harvested by fast filtration of the growth suspension through Whatman No. 1 filter paper was quick-frozen in liquid nitrogen, and stored at − 80 °C.

### Analytical methods

Glucose and ammonia in the medium, mycelial dry weigh and fatty acid methyl esters (FAME) were determined as described previously [26], and additional details are available in Additional file 6. Fatty acid-free biomass was calculated as mycelial dry weight minus total fatty acids content, total protein was determined using the bicinchoninic acid (BCA) method.

### Metabolomics and lipidomics analysis

Samples preparation and metabolites extraction were proceed as our previous described [30]. In brief, fresh biomass (50 mg) was crushed in liquid nitrogen, and then metabolites were extracted with MeOH:water (1:1) in Eppendorf tubes. Extracts were dried in a vacuum centrifuge, and then polar metabolites were analyzed using GC–MS as described, and UPLC-QE orbitrap/MS as follows. Analyses in HILIC and the RPLC mode proceeded using Waters Acquity UPLC BEH-Amide and HSS T3 columns (Waters Corp, Milford, MA, USA), respectively. Data were acquired by MS/MS via data-dependent acquisition (DDA) in the range of 70–1050 for both the positive and negative ion modes. The RAW files from GC–MS and LC-QE orbitrap/MS were generated using MSDIAL 3.7 and Compound discovery software [73, 74], respectively. The lipidomic analysis, including sample preparation, lipid extraction, and identification, proceeded as described [75]. Additional details of the metabolomic and lipidomic analyses are available in Additional file 6.

### Label-free proteomics analysis

Samples (150 mg) of cell pellets ground into a fine powder in liquid nitrogen were added to chilled 1.5-mL Eppendorf centrifuge tubes containing two clean glass beads and sonicated in 1.5 mL of 10% trichloroacetic acid/acetone containing 0.1% PMSF. The samples were vortex mixed for 60 s, then precipitated at − 20 °C overnight. The samples were vortex mixed again and separated by centrifugation at 4 °C and 15,000×*g* for 20 min. The precipitates were washed three times with acetone, dried at room temperature, lysed in denaturing lysis buffer (8 M urea, 1% SDS, 0.1% PMSF, 150 mM Tris–HCl pH 8.0) in a warm water bath for 30 min, and then separated by centrifugation at 15,000*g* for 30 min. Proteins in the supernatant were precipitated with acetone, reconstituted in UA buffer, quantified, portioned, and stored at − 80 °C. Total protein contents were quantified using the BCA method with bovine serum albumin (BSA) as the reference. Protein samples were resolved by SDS-PAGE.

The proteins were assessed using a label-free quantitative proteomic procedure as described. Purified proteins (150 μg) were diluted in UA buffer to a concentration of 1.5 μg/μL. Diluent (100 μL) was added to protein concentrators and ultrafiltration was repeated three times. The filter was washed three times with 100 μL of 50 mM NH_4_HCO_3_, then 100 μL of 50 mm DTT was added to the protein concentrators (Thermo Fisher Scientific Inc, Waltham, MA, USA) which were then placed in a water bath at 56 °C for 30 min. The filter was washed three times with 100 μL of 50 mM NH_4_HCO_3_. Next, 100 μL of a 50 mM iodoacetamide in UA buffer was added to the samples and incubated for 30 min in darkness. The filter was washed three times with 100 μL of UA buffer and three times with 100 μL of 50 mM NH_4_HCO_3_. The proteins were digested with sequencing grade trypsin (Thermo Fisher Scientific Inc.) at a final substrate/enzyme ratio of 50:1 (w/w) at 37 °C for 16 h. The digest was passed through an ultrafiltration unit (Thermo Fisher Scientific Inc.) with a molecular weight cut-off of 10 kDa, and collected peptides were assayed using a kit. The peptides in each sample were desalted on the C18 pipet-tipped columns (Thermo Fisher Scientific Inc.), concentrated by vacuum centrifugation, and reconstituted in 8 μL of 0.1% formic acid containing 2% acetonitrile for mass spectrum analysis.

Tryptic peptides were dissolved in 0.1% formic acid containing 2% acetonitrile (solvent A), directly loaded onto a custom-made reverse-phase analytical column (1.9 µm × 100 µm × 20 cm). The gradient comprised 5% to 25% solvent B (0.1% formic acid in 98% acetonitrile) over 40 min, 25% to 35% in 12 min, 35% to 80% in 4 min, hold at 80% for 4 min, at a constant flow rate of 450 nL/min on an EASY-nLC 1200 UPLC system.

The peptides were analyzed using a nanospray ionization (NSI) source followed by tandem mass spectrometry (MS/MS) in an Orbitrap Fusion Lumos (Thermo Fisher Scientific Inc.) coupled online to the UPLC. The electrospray voltage was 2.0 kV. The m/z scan range was 350 to 1550 for a full scan, and intact peptides were detected in the Orbitrap at a resolution of 60,000. Peptides were then selected for MS/MS using NCE setting 32, and the fragments were detected in the Orbitrap at a resolution of 15,000. A data-dependent procedure alternated between one MS scan followed by 20 MS/MS scans with 15.0-s dynamic exclusion. Automatic gain control (AGC) was set at 5E4. The fixed first mass was set at 100 m/z.

A protein sequence database was constructed from all predicted proteins in the *M. alpina* ATCC32222 genome (Accession no. ADAG01000000) [26]. The MS data were analyzed using MaxQuant software (version 1.6.1.0) [76, 77] and searched using our protein sequence database, MA_protein_012411.fasta, with the following parameters: minimum peptide length, 7; precursor mass tolerance, 10 ppm; fragment mass tolerance, 0.02 Da; maximum of two missed cleavages. Carbamidomethylation of cysteine was identified as a fixed modification, whereas N-acetyl protein (Protein N-term) and oxidation of methionine (Oxidation (M)) were searched as modifications included in protein quantitation. The false discovery rates (FDR) of the peptide-to-spectrum match (PSM), XPSM, protein, and site were set at 0.01. Intensity-based label-free protein quantitation proceeded using MaxQuant software with peak heights from reconstructed ion chromatograms of identified peptides. Protein abundance is represented as the total peak height of all quantified unique peptides from that protein.

### Bioinformatics and statistical analyses

Proteins or metabolites with similar change trends were grouped using k-means clustering with the Pearson correlation distance metric [54, 78–80]. Proteins or metabolites that closely correlated (*R* > 0.75) in the same cluster were included in GO-term or pathway enrichment analysis, respectively. The functional annotations (*M. alpina* ATCC32222 v1.0 database) of the proteome database of *M. alpina* ATCC32222 were achieved using EggNOG mapper 5.0 based on our previously reported genome information [26, 81]. The GO annotation results of identified proteins were extracted from the *M. alpina* ATCC32222 v1.0 database and a new GO database was constructed. Gene ontology enrichment was identified using TBtool [82] combined with the *M. alpina* ATCC32222 GO_v1.0 database. All detected metabolite and protein profiles were ranked using multivariate empirical Bayes analyses and pathway enrichment was analyzed using MetaboAnalyst 4.0 (http://www.metaboanalyst.ca/) [33, 83]. All statistical analyses and visualizations were conducted using GraphPad Prism version 6 (Graph Pad Software, San Diego, CA, U.S.A.), Adobe Illustrator CS5 (Adobe, San Jose, CA, U.S.A.), and ggplot2 package in R software.

## Supplementary information

**Additional file 1.** Additional Table S1 and Figures S1–S10.

**Additional file 2.** Qualitative and quantitative of multi-omics data.

**Additional file 3.** MEBA analysis for time-resolved metabolomics and proteomics datasets.

**Additional file 4.** Unsupervised time-resolved trend clustering analyses.

**Additional file 5.** Proteins distributed in four different organelles.

**Additional file 6.** Additional Methods.

## Data Availability

The datasets supporting the conclusions of this article are included within the article and its additional files. The mass spectrometry proteomics data have been deposited to the ProteomeXchange Consortium (http://proteomecentral.proteomexchange.org) via the iProX partner repository [84] with the dataset identifier PXD018202.

## Abbreviations

HK: Hexokinase
PK: Pyruvate kinase
G6PD: Glucose-6-phosphate 1-dehydrogenase
6GPD: Phosphogluconate dehydrogenase
IDH: Isocitrate dehydrogenase
PDH: Pyruvate dehydrogenase
ME: Malic enzyme
ACL: ATP-citrate lyase
PEPCK: Phosphoenolpyruvate carboxykinase
GPAT: Dlycero-3-phosphate acyltransferase
AGPAT: 1-acylglycerol-3-phosphate acyltransferase
DGAT: Diacylglycerol acyltransferase
PLC: Phospholipase C
PLD: Phospholipase D
ELOVL: Fatty acid elongase
FADS12: Fatty acid delta 12 desaturase
FADS6: Fatty acid delta 6 desaturase
FADS5: Fatty acid delta 5 desaturase
UBA1: Ubiquitin-activating enzyme E1
UBLE1B: Ubiquitin-like 1-activating enzyme E1 B
RPN: 26S proteasome regulatory subunit
PSMA: 20S proteasome subunit alpha
PSMB: 20S proteasome subunit beta
ltaE: Threonine aldolase
BLMH: Bleomycin hydrolase
GAD: Glutamate decarboxylase
PCD: 1-pyrroline-5-carboxylate dehydrogenase
rocD: Ornithine-oxo-acid transaminase
mTOR: Serine/threonine-protein kinase mTOR
Atg: Autophagy-related gene
CDC48: Transitional endoplasmic reticulum ATPase
SPH1: UBX domain-containing protein 1
VAC8: Vacuolar protein 8
Ypt7: GTP-Binding protein
MDH: Malate dehydrogenase
GDH: Glutamate dehydrogenase
SSADH: Succinate-semialdehyde dehydrogenase
G-6-P: d-Glucose-6-Phosphate
F-6-P: d-Fructose-6-Phosphate
PEP: Phosphoenolpyruvic acid
Pyr: Pyruvic acid
E-4-P: Erythrose-4-Phosphate
Cit: Citric acid
Aco: *cis*-aconitic acid
Suc: Succinic acid
Fum: Fumaric acid
Mal: Malic acid
Akg: Alpha-ketoglutaric acid
TAG: Triglyceride
OAA: Oxaloacetic acid
DAG: Diglyceride
PC: Phosphatidylcholine
PE: Phosphatidylethanolamine
PI: Phosphatidylinositol
PS: Phosphatidylserine
LPC: Lysophosphatidylcholine

## References

[1] Basan M, Hui S, Okano H, Zhang Z, Shen Y, Williamson JR, Hwa T (2015). Overflow metabolism in Escherichia coli results from efficient proteome allocation. Nature.

[2] Hui S, Silverman JM, Chen SS, Erickson DW, Basan M, Wang J, Hwa T, Williamson JR (2015). Quantitative proteomic analysis reveals a simple strategy of global resource allocation in bacteria. Mol Syst Biol..

[3] Dekel E, Alon U (2005). Optimality and evolutionary tuning of the expression level of a protein. Nature.

[4] Schuetz R, Zamboni N, Zampieri M, Heinemann M, Sauer U (2012). Multidimensional optimality of microbial metabolism. Science.

[5] You C, Okano H, Hui S, Zhang Z, Kim M, Gunderson CW, Wang YP, Lenz P, Yan D, Hwa T (2013). Coordination of bacterial proteome with metabolism by cyclic AMP signalling. Nature.

[6] Basan M (2018). Resource allocation and metabolism: the search for governing principles. Curr Opin Microbiol.

[7] Broach JR (2012). Nutritional control of growth and development in yeast. Genetics.

[8] Conrad M, Schothorst J, Kankipati HN, Van Zeebroeck G, Rubio-Texeira M, Thevelein JM (2014). Nutrient sensing and signaling in the yeast Saccharomyces cerevisiae. FEMS Microbiol Rev.

[9] Martin S, Parton RG (2006). Lipid droplets: a unified view of a dynamic organelle. Nat Rev Mol Cell Biol.

[10] Zechner R, Strauss JG, Haemmerle G, Lass A, Zimmermann R (2005). Lipolysis: pathway under construction. Curr Opin Lipidol.

[11] Finn PF, Dice JF (2006). Proteolytic and lipolytic responses to starvation. Nutrition..

[12] Matich EK, Ghafari M, Camgoz E, Caliskan E, Pfeifer BA, Haznedaroglu BZ, Atilla-Gokcumen GE (2018). Time-series lipidomic analysis of the oleaginous green microalga species Ettlia oleoabundans under nutrient stress. Biotechnol Biofuels.

[13] Lopez Garcia de Lomana A, Schauble S, Valenzuela J, Imam S, Carter W, Bilgin DD, Yohn CB, Turkarslan S, Reiss DJ, Orellana MV (2015). Transcriptional program for nitrogen starvation-induced lipid accumulation in Chlamydomonas reinhardtii. Biotechnol Biofuels.

[14] Pomraning KR, Kim YM, Nicora CD, Chu RK, Bredeweg EL, Purvine SO, Hu D, Metz TO, Baker SE (2016). Multi-omics analysis reveals regulators of the response to nitrogen limitation in Yarrowia lipolytica. BMC Genomics..

[15] Wang Y, Zhang S, Zhu Z, Shen H, Lin X, Jin X, Jiao X, Zhao ZK (2018). Systems analysis of phosphate-limitation-induced lipid accumulation by the oleaginous yeast Rhodosporidium toruloides. Biotechnol Biofuels.

[16] Zhu Z, Zhang S, Liu H, Shen H, Lin X, Yang F, Zhou YJ, Jin G, Ye M, Zou H, Zhao ZK (2012). A multi-omic map of the lipid-producing yeast *Rhodosporidium toruloides*. Nat Commun..

[17] Jang HD, Lin YY, Yang SS (2005). Effect of culture media and conditions on polyunsaturated fatty acids production by *Mortierella alpina*. Bioresour Technol.

[18] Ji XJ, Zhang AH, Nie ZK, Wu WJ, Ren LJ, Huang H (2014). Efficient arachidonic acid-rich oil production by *Mortierella alpina* through a repeated fed-batch fermentation strategy. Bioresour Technol.

[19] Chen B, Wan C, Mehmood MA, Chang JS, Bai F, Zhao X (2017). Manipulating environmental stresses and stress tolerance of microalgae for enhanced production of lipids and value-added products—A review. Bioresour Technol.

[20] Anand J, Arumugam M (2015). Enhanced lipid accumulation and biomass yield of *Scenedesmus quadricauda* under nitrogen starved condition. Bioresour Technol.

[21] Liu T, Li Y, Liu F, Wang C (2016). The enhanced lipid accumulation in oleaginous microalga by the potential continuous nitrogen-limitation (CNL) strategy. Bioresour Technol.

[22] Arora N, Pienkos PT, Pruthi V, Poluri KM, Guarnieri MT (2018). Leveraging algal omics to reveal potential targets for augmenting TAG accumulation. Biotechnol Adv.

[23] Martien JI, Amador-Noguez D (2017). Recent applications of metabolomics to advance microbial biofuel production. Curr Opin Biotechnol.

[24] Higashiyama K, Yaguchi T, Akimoto K, Fujikawa S, Shimizu S (1998). Enhancement of arachidonic acid production by *Mortierella alpina* 1S-4. J Am Oil Chem Soc.

[25] Ji XJ, Ren LJ, Nie ZK, Huang H, Ouyang PK (2014). Fungal arachidonic acid-rich oil: research, development and industrialization. Crit Rev Biotechnol.

[26] Wang L, Chen W, Feng Y, Ren Y, Gu Z, Chen H, Wang H, Thomas MJ, Zhang B, Berquin IM (2011). Genome characterization of the oleaginous fungus *Mortierella alpina*. PLoS ONE.

[27] Li X, Lin Y, Chang M, Jin Q, Wang X (2015). Efficient production of arachidonic acid by *Mortierella alpina* through integrating fed-batch culture with a two-stage pH control strategy. Bioresour Technol.

[28] Tang X, Chen H, Mei T, Ge C, Gu Z, Zhang H, Chen YQ, Chen W (2018). Characterization of an omega-3 desaturase from *Phytophthora parasitica* and application for eicosapentaenoic acid production in *Mortierella alpina*. Front Microbiol..

[29] Chen H, Hao G, Wang L, Wang H, Gu Z, Liu L, Zhang H, Chen W, Chen YQ (2015). Identification of a critical determinant that enables efficient fatty acid synthesis in oleaginous fungi. Sci Rep.

[30] Lu H, Chen H, Tang X, Yang Q, Zhang H, Chen YQ, Chen W (2019). Evaluation of metabolome sample preparation and extraction methodologies for oleaginous filamentous fungi *Mortierella alpina*. Metabolomics.

[31] Klug L, Daum G (2014). Yeast lipid metabolism at a glance. FEMS Yeast Res.

[32] Alipanah L, Rohloff J (2015). Whole-cell response to nitrogen deprivation in the diatom *Phaeodactylum tricornutum*. J Exp Bot.

[33] Tai YC, Speed TP (2006). A multivariate empirical Bayes statistic for replicated microarray time course data. Ann Stat.

[34] Rubinsztein DC, Marino G, Kroemer G (2011). Autophagy and aging. Cell.

[35] Chen X, Williams C (2018). Fungal peroxisomes proteomics. Subcell Biochem.

[36] Dong HP, Williams E, Wang DZ, Xie ZX, Hsia RC, Jenck A, Halden R, Li J, Chen F, Place AR (2013). Responses of *Nannochloropsis oceanica* IMET1 to long-term nitrogen starvation and recovery. Plant Physiol.

[37] Yao L, Shen H, Wang N, Tatlay J, Li L, Tan TW, Lee YK (2017). Elevated acetyl-CoA by amino acid recycling fuels microalgal neutral lipid accumulation in exponential growth phase for biofuel production. Plant Biotechnol J.

[38] Fan J, Andre C, Xu C (2011). A chloroplast pathway for the de novo biosynthesis of triacylglycerol in *Chlamydomonas reinhardtii*. FEBS Lett.

[39] Bates PD (2016). Understanding the control of acyl flux through the lipid metabolic network of plant oil biosynthesis. Biochim Biophys Acta.

[40] Allen AE, Dupont CL, Obornik M, Horak A, Nunes-Nesi A, McCrow JP, Zheng H, Johnson DA, Hu H, Fernie AR, Bowler C (2011). Evolution and metabolic significance of the urea cycle in photosynthetic diatoms. Nature.

[41] Smith SR, Dupont CL, McCarthy JK (2019). Evolution and regulation of nitrogen flux through compartmentalized metabolic networks in a marine diatom. Nat Commun.

[42] Ye C, Xu N, Chen H, Chen YQ, Chen W, Liu L (2015). Reconstruction and analysis of a genome-scale metabolic model of the oleaginous fungus *Mortierella alpina*. BMC Syst Biol.

[43] Liang Y, Kong F (2019). Branched-chain amino acid catabolism impacts triacylglycerol homeostasis in *Chlamydomonas reinhardtii*. Plant Physiol.

[44] Huo YX, Cho KM, Rivera JG, Monte E, Shen CR, Yan Y, Liao JC (2011). Conversion of proteins into biofuels by engineering nitrogen flux. Nat Biotechnol.

[45] Vorapreeda T, Thammarongtham C, Cheevadhanarak S, Laoteng K (2012). Alternative routes of acetyl-CoA synthesis identified by comparative genomic analysis: involvement in the lipid production of oleaginous yeast and fungi. Microbiology.

[46] Levitan O, Dinamarca J, Zelzion E, Lun DS, Guerra LT, Kim MK, Kim J, Van Mooy BA, Bhattacharya D, Falkowski PG (2015). Remodeling of intermediate metabolism in the diatom *Phaeodactylum tricornutum* under nitrogen stress. Proc Natl Acad Sci USA..

[47] Castillo A, Taboada H, Mendoza A, Valderrama B, Encarnacion S, Mora J (2000). Role of GOGAT in carbon and nitrogen partitioning in *Rhizobium etli*. Microbiology.

[48] Yu LJ, Qin WM, Lan WZ, Zhou PP, Zhu M (2003). Improved arachidonic acids production from the fungus *Mortierella alpina* by glutamate supplementation. Bioresour Technol.

[49] Lan WZ, Qin WM, Yu LJ (2002). Effect of glutamate on arachidonic acid production from *Mortierella alpina*. Lett Appl Microbiol.

[50] Hao G, Chen H, Wang L, Gu Z, Song Y, Zhang H, Chen W, Chen YQ (2014). Role of malic enzyme during fatty acid synthesis in the oleaginous fungus *Mortierella alpina*. Appl Environ Microbiol.

[51] Ge F, Huang W, Chen Z, Zhang C, Xiong Q, Bowler C, Yang J, Xu J, Hu H (2014). Methylcrotonyl-CoA carboxylase regulates triacylglycerol accumulation in the model diatom *Phaeodactylum tricornutum*. Plant Cell..

[52] Crown SB, Marze N, Antoniewicz MR (2015). Catabolism of branched chain amino acids contributes significantly to synthesis of odd-chain and even-chain fatty acids in 3T3-L1 adipocytes. PLoS ONE.

[53] Wang F, Bi Y, Diao J, Lv M, Cui J, Chen L, Zhang W (2019). Metabolic engineering to enhance biosynthesis of both docosahexaenoic acid and odd-chain fatty acids in *Schizochytrium* sp. S31. Biotechnol Biofuels.

[54] Zienkiewicz A, Zienkiewicz K, Poliner E, Pulman JA, Du ZY, Stefano G, Tsai CH, Horn P, Feussner I, Farre EM (2020). The microalga nannochloropsis during transition from quiescence to autotrophy in response to nitrogen availability. Plant Physiol.

[55] Hao G, Chen H, Yang B, Du K, Wang H, Gu Z, Zhang H, Chen W, Chen YQ (2016). Substrate specificity ofMortierella alpinaΔ9-III fatty acid desaturase and its value for the production of omega-9 MUFA. Eur J Lipid Sci Technol.

[56] Berner N, Reutter KR, Wolf DH (2018). Protein quality control of the endoplasmic reticulum and ubiquitin-proteasome-triggered degradation of aberrant proteins: yeast pioneers the path. Annu Rev Biochem.

[57] Varshavsky A (2017). The ubiquitin system, autophagy, and regulated protein degradation. Annu Rev Biochem.

[58] Zheng N, Shabek N (2017). Ubiquitin ligases: structure, function, and regulation. Annu Rev Biochem.

[59] Dikic I (2017). Proteasomal and autophagic degradation systems. Annu Rev Biochem.

[60] Pollack JK, Harris SD, Marten MR (2009). Autophagy in filamentous fungi. Fungal Genet Biol.

[61] Russell RC, Yuan HX, Guan KL (2014). Autophagy regulation by nutrient signaling. Cell Res.

[62] Masclaux-Daubresse C, Chen Q, Have M (2017). Regulation of nutrient recycling via autophagy. Curr Opin Plant Biol.

[63] Singh R, Kaushik S, Wang Y, Xiang Y, Novak I, Komatsu M, Tanaka K, Cuervo AM, Czaja MJ (2009). Autophagy regulates lipid metabolism. Nature.

[64] Couso I, Perez-Perez ME, Martinez-Force E, Kim HS, He Y, Umen JG, Crespo JL (2018). Autophagic flux is required for the synthesis of triacylglycerols and ribosomal protein turnover in Chlamydomonas. J Exp Bot.

[65] Fan J, Yu L, Xu C (2019). Dual role for autophagy in lipid metabolism in *Arabidopsis*. Plant Cell..

[66] Kajikawa M, Yamauchi M, Shinkawa H, Tanaka M, Hatano K, Nishimura Y, Kato M, Fukuzawa H (2019). Isolation and characterization of chlamydomonas autophagy-related mutants in nutrient-deficient conditions. Plant Cell Physiol.

[67] Elander PH, Minina EA, Bozhkov PV (2018). Autophagy in turnover of lipid stores: trans-kingdom comparison. J Exp Bot.

[68] Krick R, Bremer S, Welter E, Schlotterhose P, Muehe Y, Eskelinen EL, Thumm M (2010). Cdc48/p97 and Shp1/p47 regulate autophagosome biogenesis in concert with ubiquitin-like Atg8. J Cell Biol.

[69] Chen H, Hu J, Qiao Y, Chen W, Rong J, Zhang Y, He C, Wang Q (2015). Ca(2 +)-regulated cyclic electron flow supplies ATP for nitrogen starvation-induced lipid biosynthesis in green alga. Sci Rep..

[70] McGrath JP, Jentsch S, Varshavsky A (1991). UBA 1: an essential yeast gene encoding ubiquitin-activating enzyme. EMBO J.

[71] Zhang YM, Chen H, He CL, Wang Q (2013). Nitrogen starvation induced oxidative stress in an oil-producing green alga *Chlorella sorokiniana* C3. PLoS ONE.

[72] Yoon I, Nam M, Kim H, Moon H, Kim S, Jang J, Song J, Jeong S, Kim S, Cho S (2020). Glucose-dependent control of leucine metabolism by leucyl-tRNA synthetase 1. Science.

[73] Hao L, Wang J, Page D, Asthana S, Zetterberg H, Carlsson C, Okonkwo OC, Li L (2018). Comparative evaluation of ms-based metabolomics software and its application to preclinical Alzheimer’s disease. Sci Rep..

[74] Lai Z, Tsugawa H (2018). Identifying metabolites by integrating metabolome databases with mass spectrometry cheminformatics. Nat Methods.

[75] Lu H, Chen H, Tang X, Yang Q, Zhang H, Chen YQ, Chen W (2019). Ultra performance liquid chromatography-Q exactive orbitrap/mass spectrometry-based lipidomics reveals the influence of nitrogen sources on lipid biosynthesis of *Mortierella alpina*. J Agri Food Chem.

[76] Tyanova S, Temu T, Cox J (2016). The MaxQuant computational platform for mass spectrometry-based shotgun proteomics. Nat Protoc.

[77] Yu Y, Zhang L, Li T, Wu N, Jiang L, Ji X, Huang H (2018). How nitrogen sources influence Mortierella alpina aging: from the lipid droplet proteome to the whole-cell proteome and metabolome. J Proteomics..

[78] Gu Z, Eils R, Schlesner M (2016). Complex heatmaps reveal patterns and correlations in multidimensional genomic data. Bioinformatics.

[79] Hejblum BP, Skinner J, Thiebaut R (2015). Time-course gene set analysis for longitudinal gene expression data. PLoS Comput Biol.

[80] Gong T, Zhang C, Ni X, Li X, Li J, Liu M, Zhan D, Xia X, Song L, Zhou Q (2020). A time-resolved multi-omic atlas of the developing mouse liver. Genome Res.

[81] Huerta-Cepas J, Szklarczyk D, Heller D, Hernandez-Plaza A, Forslund SK, Cook H, Mende DR, Letunic I, Rattei T, Jensen LJ (2019). eggNOG 5.0: a hierarchical, functionally and phylogenetically annotated orthology resource based on 5090 organisms and 2502 viruses. Nucleic Acids Res.

[82] Chen C, Chen H, Zhang Y (2020). TBtools - an integrative toolkit developed for interactive analyses of big biological data. Mol Plant..

[83] Spicer R, Salek RM (2017). Navigating freely-available software tools for metabolomics analysis. Metabolomics.

[84] Ma J, Chen T, Wu S, Yang C, Bai M, Shu K, Li K, Zhang G, Jin Z, He F (2019). iProX: an integrated proteome resource. Nucleic Acids Res.

