# Supplementary material for: Time-resolved multi-omics analysis reveals the role of nutrient stress-induced resource reallocation for TAG accumulation in oleaginous fungus Mortierella alpina
Source: Biotechnol Biofuels. 2020 Jul 1;13:116. doi: 10.1186/s13068-020-01757-1 (PMC7328260; doi:10.1186/s13068-020-01757-1)
Supplement: Supplementary file 1 — Additional file 1. Additional Table S1 and Figures S1–S10. [file 13068_2020_1757_MOESM1_ESM.docx]

**Table S1. Percentage change of fatty acid profiles during the fermentation process.**

| **Fatty acids(%)** | **24 h** | **48 h** | **96 h** | **144 h** | **168 h** | **216 h** |
| --- | --- | --- | --- | --- | --- | --- |
| **C14:0** | 1.26±0.11 | 1.21±0.08 | 0.89±0.01 | 0.57±0.01 | 0.48±0.01 | 0.41±0.01 |
| **C16:0** | 15.98±0.14 | 14.64±0.09 | 15.53±0.18 | 14.11±0.12 | 13.39±0.05 | 13.33±0.11 |
| **C18:0** | 12.94±0.36 | 14.34±0.19 | 13.48±0.01 | 14.24±0.06 | 14.535±0.16 | 14.63±0.04 |
| **C18:1** | 19.82±0.23 | 32.78±0.01 | 24.15±0.16 | 23.23±0.13 | 23.405±0.31 | 23.1±0.14 |
| **C18:2** | 5.25±0.01 | 4.52±0.11 | 10.45±0.19 | 11.63±0.12 | 11.14±0.20 | 11.2±0.1 |
| **C18:3** | 4.62±0.16 | 2.82±0.01 | 3.68±0.08 | 3.68±0.06 | 3.555±0.08 | 3.52±0.07 |
| **C20:0** | 1.52±0.06 | 1.29±0.04 | 1.2±0.08 | 1.23±0.03 | 0.95±0.02 | 0.92±0.02 |
| **C20:1** | 0.48±0.06 | 0.87±0.11 | 0.88±0.05 | 0.93±0.01 | 1.035±0.06 | 0.81±0.01 |
| **C20:4** | 32.15±0.66 | 23.11±0.35 | 24.57±0.021 | 24.86±0.12 | 24.26±0.48 | 24.31±0.13 |
| **C22:0** | 1.37±0.01 | 1.48±0.04 | 1.35±0.06 | 1.31±0.04 | 1.32±0.08 | 1.31±0.03 |
| **C22:3** | 4.55±0.03 | 2.58±0.01 | 3.31±0.01 | 3.6±0.01 | 3.595±0.08 | 3.534±0.01 |

Fig. S1


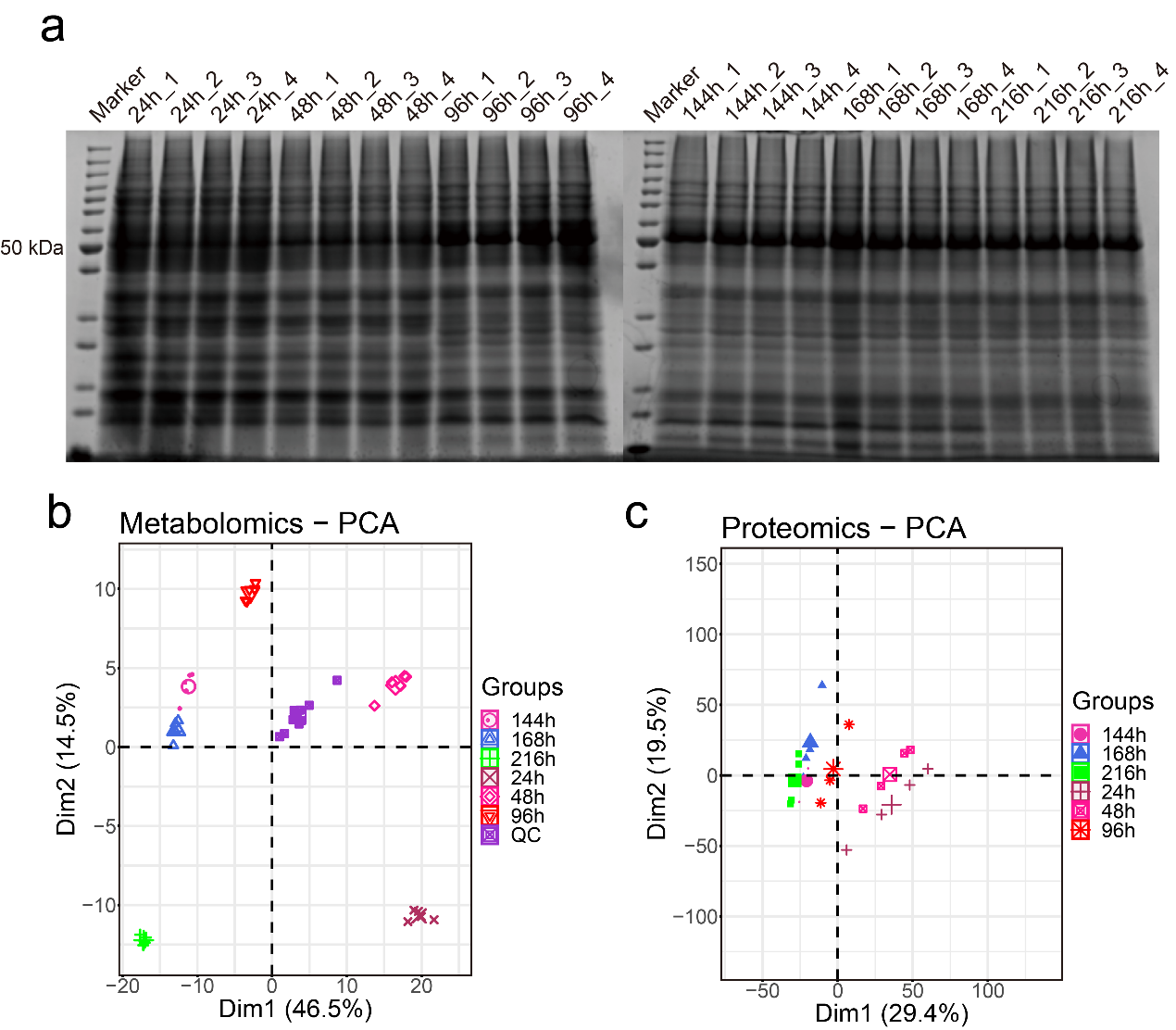


Additional_Fig_S1:

**a)**. SDS-PAGE analysis of the whole cell proteins in different time point. Four repeats were proceed for each time point; **b-c).** PCA of 24, 48, 96, 144, 168 and 216 h samples for the corresponding metabolomics and proteomics datasets.,

Fig. S2


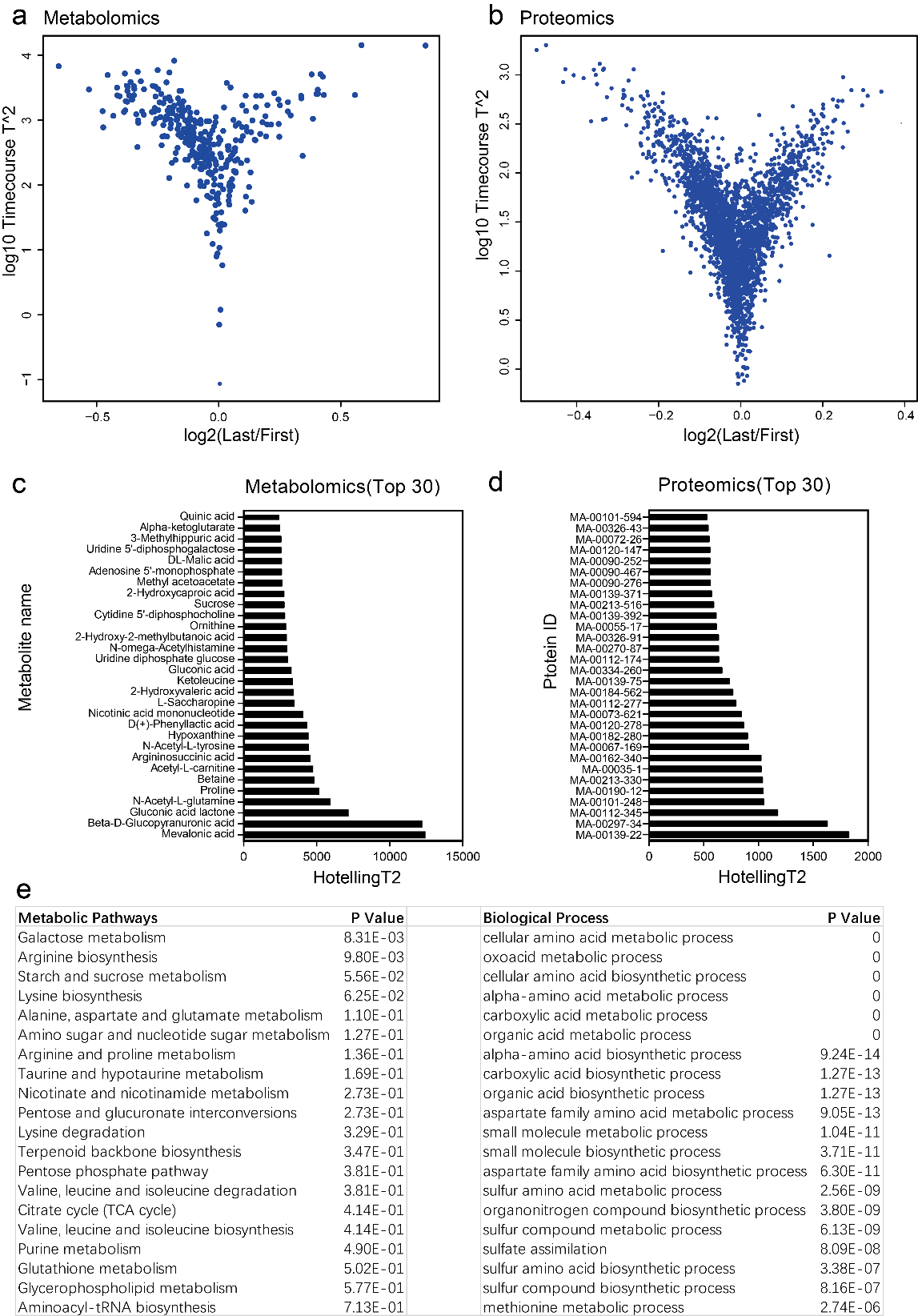


Additional_Fig_S2: Multivariate empirical Bayes analyses (MEBA) analysis for time-resolved metabolomics and proteomics datasets.

a-b). Distribution of Hotelling T2 values for identified metabolite and protein profiles from Multivariate Empirical Bayes analyses analysis.

c-d). Top 30 metabolites and proteins ranked based on Hotelling T2 values.

e). Pathways enrichment and GO enrichment results by using the top 30 Hotelling T2 features from Multivariate Empirical Bayes Analyses.

Fig. S3


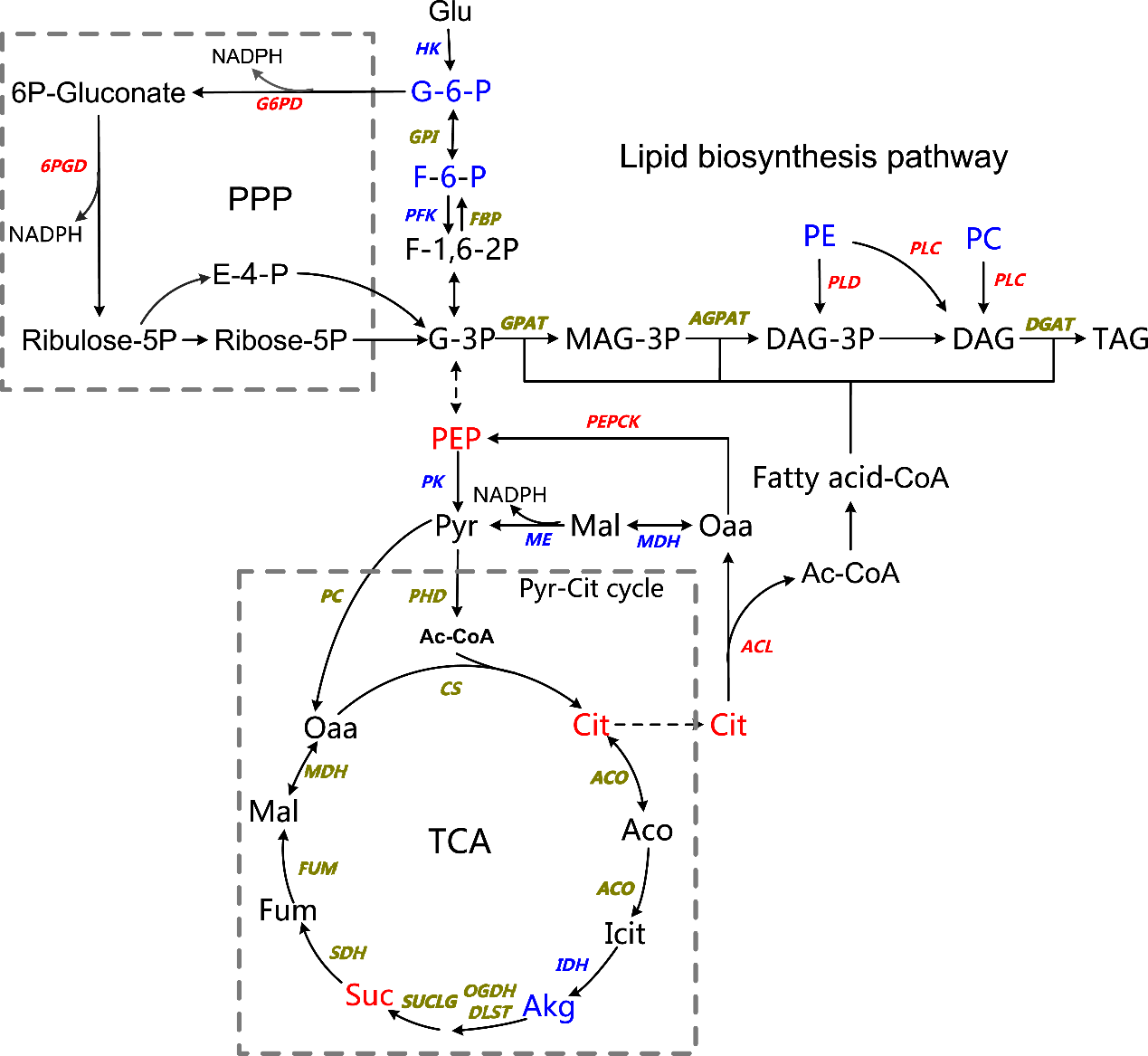


Additional_Fig_S3: Changes of proteins and metabolites involved in carbon metabolism and lipid metabolism during the nitrogen deprivation induced TAGs accumulation. Up-regulated metabolites and enzymes are plotted in red and down-regulated metabolites and enzymes in blue.

Fig. S4





Additional_Fig_S4: Change of enzymes involved in carbon metabolism during nitrogen stress-induced TAGs accumulation in the oleaginous fungi *M. alpina*.

Fig. S5


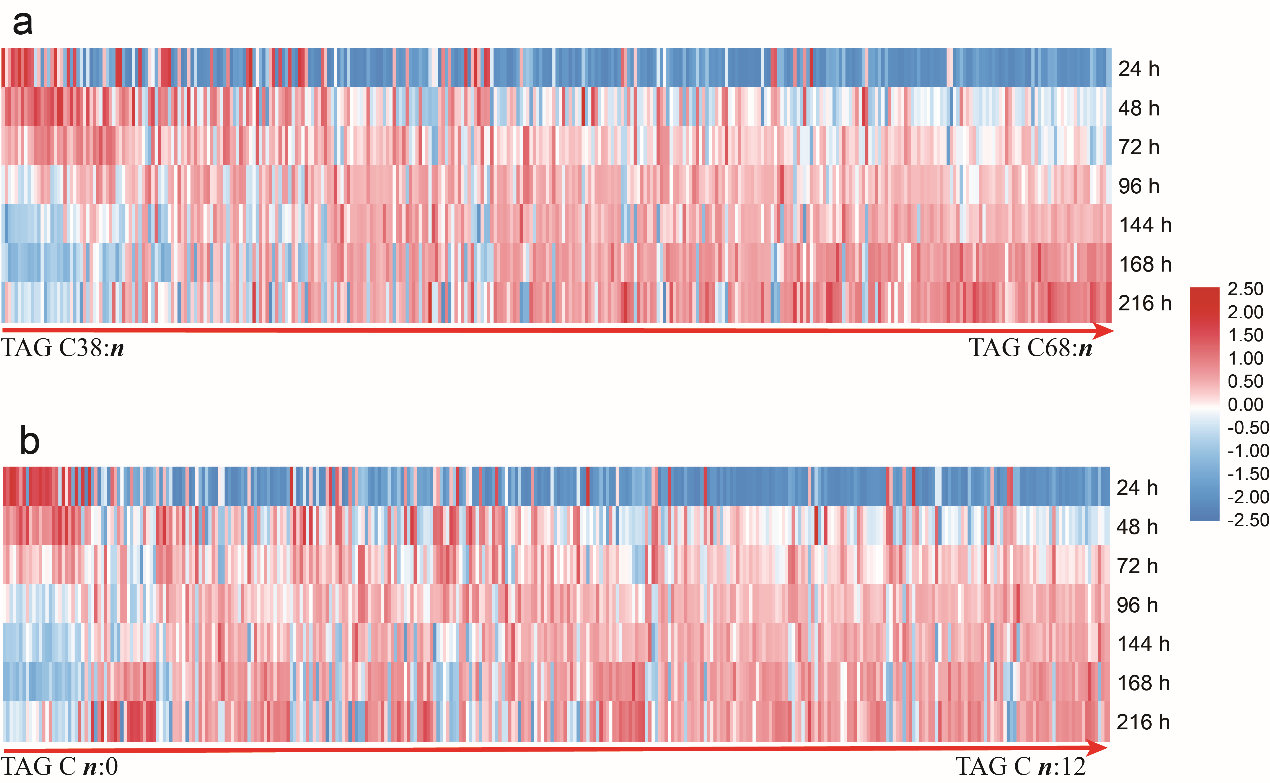


Additional_Fig_S5: Change of TAG profiles ranking based on their chain length (a) and number of unsaturated bonds (b).

Fig. S6


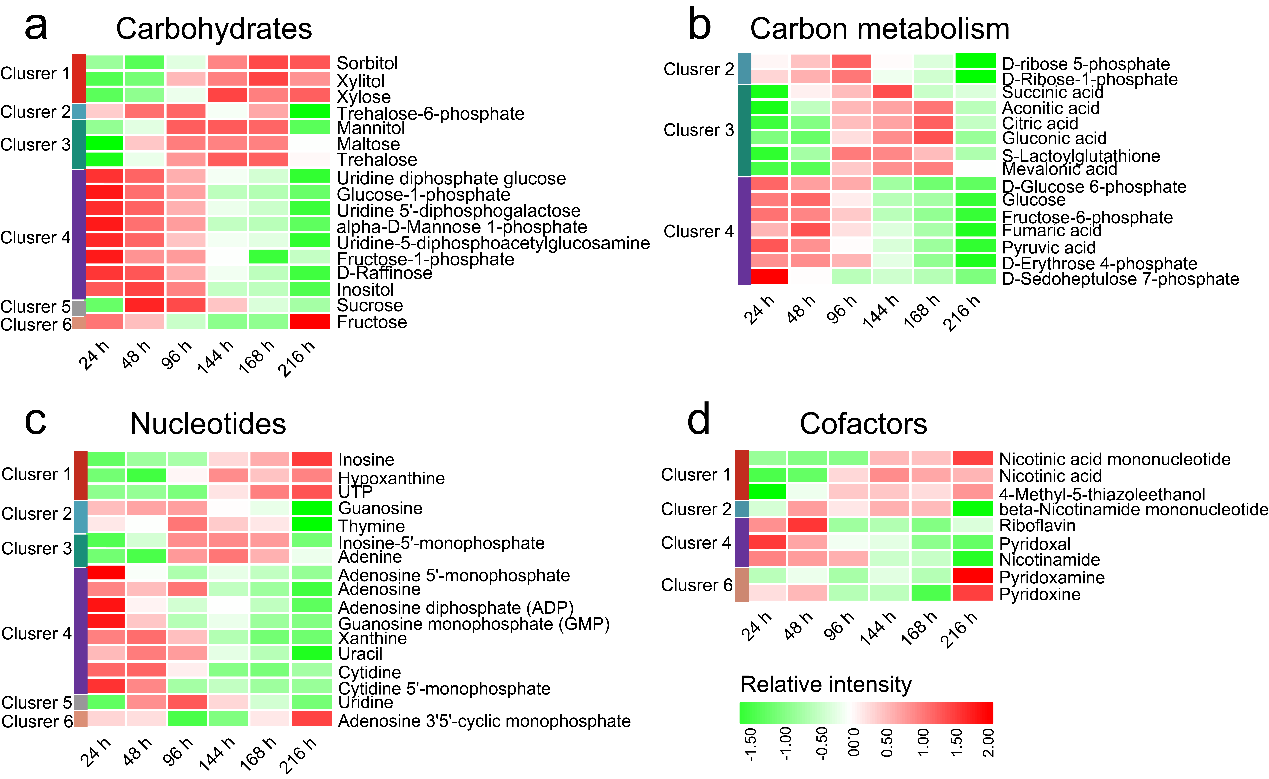


Additional_Fig_S6: Heatmap representation for intensity changes of post-filtered set (R > 0.75) of metabolites in each metabolic modules (functional/pathway clusters).

Fig. S7


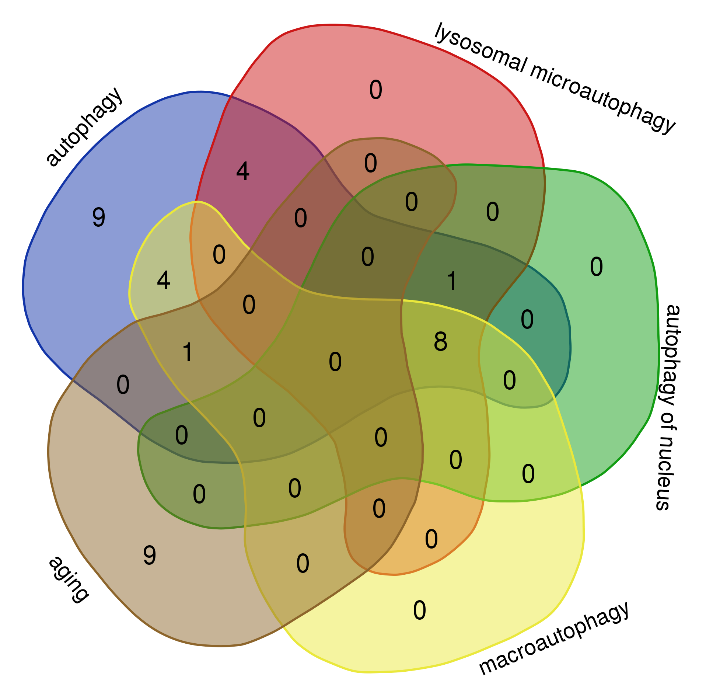


Additional_Fig_S7: Venn diagram showing the overlap in the numbers of proteins among different autophagy items (included aging).

Fig. S8





Additional_Fig_S8: The effects of GABA shunt and amino acid metabolism on the fatty acid biosynthesis.

a-c). change of enzymes involved in GABA shunt and amino acid degradation. d). the effects of L-valine supplementation on the biosynthesis of odd fatty acids. GDH, Glutamate dehydrogenase., GAD, Glutamate decarboxylase., SSADH, Succinic semialdehyde dehydrogenase.

Fig. S9


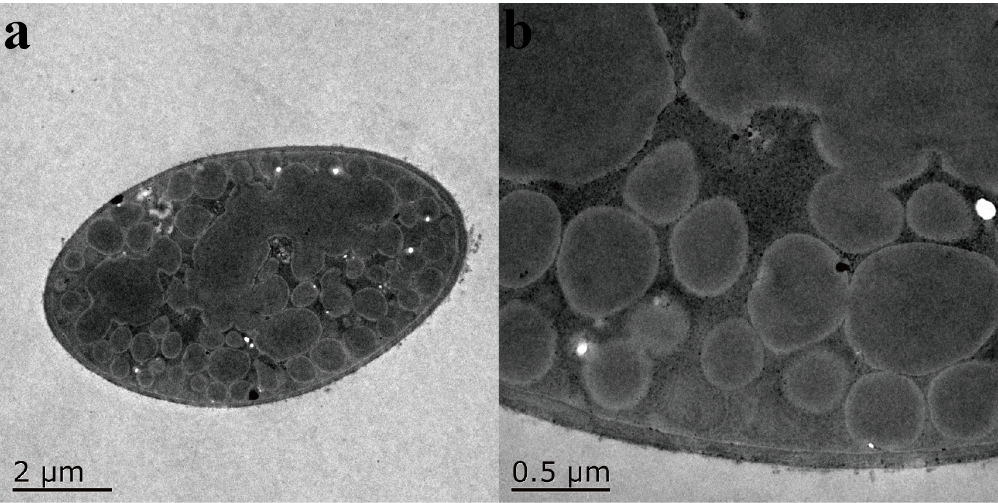


Additional_Fig_S9: Transmission electron microscope images of mycelia at the end of the fermentation process (168 h).

Fig. S10

**
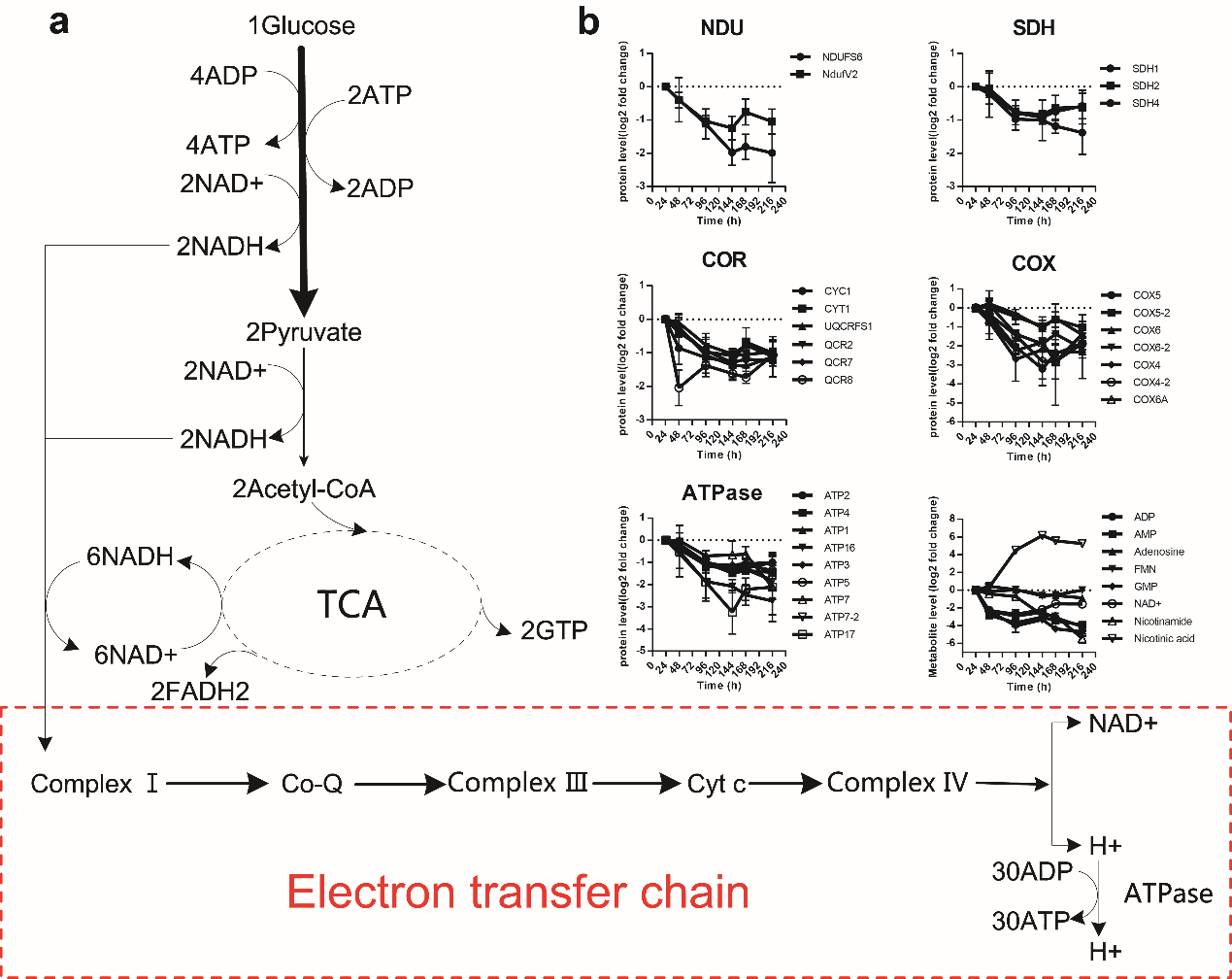
**

Additional_Fig_S10: Down-regulation of oxidative phosphorylation during nitrogen stress-induced TAGs accumulation in the oleaginous fungi *M. alpina*.

Based on the time-course GO enrichment results, ATP metabolism was significantly changed under nitrogen deprivation (Fig. 2e, cluster 2). We selected the enzymes of which involved in oxidative phosphorylation from cluster 2. All kind of enzymes including NADH/succinate dehydrogenase (NDU), cytochrome c reductase/oxidase (COR/COX) and ATPase respiratory involved in electron transport chain were rapidly decreased when the nitrogen source was exhausted. These results mean that the ATP synthesis was extremely blocked in nitrogen deprivation condition. The decrease of ADP and AMP level in the fermentation process also verified this conclusion (Fig. S10b). All these results indicate the repression of oxidative phosphorylation activity under nitrogen deprivation.
