# Supplementary material for: Time-resolved multi-omics analysis reveals the role of nutrient stress-induced resource reallocation for TAG accumulation in oleaginous fungus Mortierella alpina
Source: Biotechnol Biofuels. 2020 Jul 1;13:116. doi: 10.1186/s13068-020-01757-1 (PMC7328260; doi:10.1186/s13068-020-01757-1)
Supplement: Supplementary file 6 — Additional file 6. Additional Methods. [file 13068_2020_1757_MOESM6_ESM.docx]

**Additional_Methods**

**Cultivation of the** [***M.***](javascript:;) [***alpina***](javascript:;)

[*M.*](javascript:;) [*alpina*](javascript:;) cultivation was were performed as described previously [[1](#_ENREF_1), [2](#_ENREF_2)]. *M. alpina* ATCC32222 was maintained on GY agar slants (per litre: 30.0 g of glucose, 5.0 g of yeast extract, 2.0 g of KNO_3_, 1.5 g of MgSO_4_.7H_2_O, 1.0 g of NaH_2_PO_4_) at 4°C. *M. alpina* mycelium was transferred from the GY agar slant into 100 mL of GY seed media in a 250 mL flask to inoculate the seed culture and shaken at 200 rpm and 28 °C for 2 days. The mycelia were collected and homogenized in fresh seed medium using a Braun hand blender (IKA, Germany). A 1 mL aliquot of the mycelial suspension was inoculated in 100 mL of seed media and shaken for 36 h at 200 rpm and 28 °C. The mycelia were collected, and the above procedures were repeated. Then, 1% (v/v) of the seed culture was inoculated into 100 mL of the fermentation broth (Kendrick broth, per litre: 30.0 g of glucose, 2.0 g of diammonium tartrate, 7.0 g of KH_2_PO_4_, 2.0 g of Na_2_HPO_4_, 1.5 g of MgSO_4_.7H_2_O, 1.5 g of yeast extract, 0.1 g of CaCl_2_·2H_2_O, 8.0 mg of FeCl3·6H_2_O, 1 mg of ZnSO_4_·7H_2_O, 0.1 mg of CuSO_4_·5H_2_O, 0.1mg of Co(NO_3_)_2_·6H_2_O and 0.1mg of MnSO_4_·5H_2_O, pH 6.0) in a 250 mL flask and shaken at 200 rpm and 28°C for 36 h used as the final seed. The final seed were transferred into a 7.5 L fermentation tank containing 4.0 L of fermentation broth and cultured for 216 h at 28 °C, pH 6.0 with agitation rate at 300 rpm, the aeration rate at 1.0 vvm.

**Targeted Analysis of Fatty Acid Profiles**

Total fatty acid extraction and methyl ester analysis were performed as described previously [[3](#_ENREF_3)]. Briefly, approximately 50 mg of freeze-dried mycelia was broken down by repeated freeze−thaw cycles under acidified conditions and extracted using MeOH/chloroform (1:1). Pentadecanoic acid was added as an internal standard. Fatty acids were converted to methyl esters using 10% hydrochloric acid methanol. The fatty acid profiles were analyzed using gas chromatography-mass spectrometry (GC-MS, QP2010, Shimadzu Co., Kyoto, Japan). The DB-WAXetr column (30 m × 0.32 mm; film thickness, 0.25 μm) was used. The derivatized sample (1 μL) was injected into the GC-MS system in split mode (split ratio of 10:1). The oven temperature was held at 150 °C for 3 min and then increased to 190 °C (at 10 °C/min) and subsequently to 220 °C (at 5 °C/min) for 16 min. MS was operated in a scan range of m/z 50-550. The ion source temperature was 220 °C. The electron ionization was operated at 70 eV. Fatty acid methyl esters (FAMEs) were identified through comparison to commercial FAME standards (GLC-463, Nu-Chek, Elysian, MN, U.S.A.).

**UPLC-Q-Exactive Orbitrap/MS-Based Metabolomics and lipidomics analysis**

Mycelia were collected by fast vacuum filtration using a paper filter (Whatman no. 1) and washed with 4°C 0.9% NaCl at room temperature. After quick-freezing in liquid nitrogen, the frozen cell pellets were further ground into powder (fresh biomass) and stored at -80°C for further use [[2](#_ENREF_2)].

For metabolomics analysis, metabolites extraction was done as our previous described[[2](#_ENREF_2)]. For Lipidomics analysis, lipids extraction followed the SOP of Fiehn Lab [[4](#_ENREF_4), [5](#_ENREF_5)]. Briefly, The fresh biomass was ground into fine powder. 50.00 mg ground powder were was added into a pre-chilled centrifuge tube (1.5 mL, Eppendorf) containing two clean glass beads. The ground powder was sonicated in water bath for 5 min with 225µL cold methanol and then vortexed for 10 s. Next, 750µL cold MTBE was sequentially added and vortexed for 10 s. The solvent mixture was shaken for 30 min at 4°C. Finally, 188 µL Milli-Q Ultrapure water was added to induce phase separation. The samples were vortexed and centrifuged at 4°C, 12,000 rpm for 15 min. The upper phase was transferred to fresh tube and dried in a vacuum centrifuge. The dried samples were re-suspend in 110 µL chloroform:Methanol:water (60:30:4.5,v/v/v) for LC/MSMS analysis.

The untargeted metabolomics and lipidomic analysis were conducted using a Dionex UltiMate 3000 UPLC system (Santa Clara, CA, USA) coupled to a HESI probe with a Q-Exactive Orbitrap mass spectrometer (Thermo Fisher, CA, USA) [[3](#_ENREF_3)].

The metabolomics extracted from *M. alpina* were separated on a Waters Acquity UPLC BEH Amide column (100 × 2.1 mm, 1.7 μm) and HSS T3 column (100 × 2.1 mm, 1.7 μm) for HILIC and RPLC model, respectively, maintained at 30°C. All MS experiments were performed in positive and negative ion modes. For HILIC, the mobile phase of positive model was consisted of A (10mM HCOONH_4_, 0.1% formic acid, ACN:H_2_O 95:5) and B(10mM HCOONH_4_, 0.1% formic acid, ACN:H_2_O 50:50) using a gradient elution of 98% A at 0-1 min, 98%-50% A at 1-19 min, 50% A at 19-22 min, 50%-98% A at 22-23 min, 98% A at 23-28 min., For mobile phase of negative model, replace 0.1% formic acid with ammonia and control pH at 9.0. For RPLC, the mobile phase was consisted of A (0.1% formic acid -H_2_O) and B(ACN) using a gradient elution of 98% A at 0-1 min, 98%-2% A at 1-12 min, 98% A at 12-15 min, 98%-2% A at 15-15.1 min, 98% A at 15.1-20 min. The injection volume and flow rate was set at 2 µL and 0.3 mL/min, respectively.

The lipids extracted from *M. alpina* were separated on a Waters Acquity UPLC BEH C18 column (100 × 2.1 mm, 1.7 μm) maintained at 55°C. All MS experiments were performed in positive modes. The mobile phase was consisted of A (60:40 ACN:Water containing 10 mM ammonium formate and 0.1% formic acid) and B (90:10 IPA:ACN containing 10 mM ammonium formate and 0.1% formic acid) using a gradient elution of 0-70% B at 1-3min, 70-95% B at 3-26 min, 95-0% B at 26-27 min, 0% B at 27-30 min. The injection volume and flow rate was set at 2 µL and 0.25mL/min, respectively.

The data were acquired with data dependent MS/MS acquisition at ranges of 70-1050 and 200-1200 for metabolomics and lipidomics, respectively. The resolution of full scan and fragment spectra were collected at 70,000 and 17,500, respectively. The applied source and ion transfer parameters were as followed: spray voltage 3.5 kV (positive) and 2.8 kV (negative). For both ionization modes, the sheath gas, aux gas, the capillary temperature and the heater temperature were maintained at 35, 15 (arbitrary units), 325◦C and 300◦C, respectively. The S-Lens RF level was set at 50. For full-MS, AGC target was set as 3e6, max injection time was set as 100 ms. For ddMS2, AGC was target set as 1e5, max injection time was set as 80 ms. The data-dependent MS2 mode TopN: 10, normalized collision energy for metabolomics: 20, 40 and 60., normalized collision energy for lipidomics: 25 and 30; isolation window: 1.5 m/z.

Compound dissolve 2.0 (CD software, Thermo, USA) and MS-DIAL 3.70 software were used for “Raw files” processing and metabolites identification [[6](#_ENREF_6)]. For CD software, metabolite identification was achieved by searching MS and MS/MS data against mzCloud database (<https://www.mzcloud.org>). For MS-DIAL software, the “raw” format files were also converted to “ABF” format using an ABF converter. MS-DIAL 3.70 equipped with the Fiehn HILIC (HILIC) and MassBank (RPLC) database was used for peak exaction, retention time adjustment, peak alignment, deconvolution analysis, and identification. Lipid identification were performed using MS-DIAL 3.70. LipidMsmsBinaryDB-VS46-FiehnO database was used for lipids identification, parameter settings were as follows. Data collection: MS1/MS2 tolerance, 0.01/0.05 Da; minimum peak height, 20000 amplitude. Identification: accurate mass tolerance MS1/MS2, 0.01/0.05 Da., score cut off, 80%. Alignment: retention time tolerance, 0.05 min, MS1 tolerance, 0.015 Da. All of the annotations produced by Compound discover and MS-DIAL software were checked and combined manually.

**References**

1. Wang L, Chen W, Feng Y, Ren Y, Gu Z, Chen H, Wang H, Thomas MJ, Zhang B, Berquin IM: Genome characterization of the oleaginous fungus Mortierella alpina. PLoS ONE. 2011; 6:e28319-.

2. Lu H, Chen H, Tang X, Yang Q, Zhang H, Chen YQ, Chen W: Evaluation of metabolome sample preparation and extraction methodologies for oleaginous filamentous fungi Mortierella alpina. Metabolomics. 2019; 15.

3. Lu H, Chen H, Tang X, Yang Q, Zhang H, Chen YQ, Chen W: Ultra Performance Liquid Chromatography-Q Exactive Orbitrap/Mass Spectrometry-Based Lipidomics Reveals the Influence of Nitrogen Sources on Lipid Biosynthesis of Mortierella alpina. Journal Of Agricultural And Food Chemistry. 2019; 67:10984-10993.

4. Matyash V, Liebisch G, Kurzchalia TV, Shevchenko A, Schwudke D: Lipid extraction by methyl-tert-butyl ether for high-throughput lipidomics. J Lipid Res. 2008; 49:1137-1146.

5. Cajka T, Fiehn O: Comprehensive analysis of lipids in biological systems by liquid chromatography-mass spectrometry. Trends Analyt Chem. 2014; 61:192-206.

6. Hao L, Wang J, Page D, Asthana S, Zetterberg H, Carlsson C, Okonkwo OC, Li L: Comparative Evaluation of MS-based Metabolomics Software and Its Application to Preclinical Alzheimer's Disease. Sci Rep. 2018; 8:9291.
